# Forecasting Global Fire Emissions on Subseasonal to Seasonal (S2S) Time Scales

**DOI:** 10.1029/2019MS001955

**Published:** 2020-09-18

**Authors:** Yang Chen, James T. Randerson, Shane R. Coffield, Efi Foufoula‐Georgiou, Padhraic Smyth, Casey A. Graff, Douglas C. Morton, Niels Andela, Guido R. van der Werf, Louis Giglio, Lesley E. Ott

**Affiliations:** ^1^ Department of Earth System Science University of California Irvine CA USA; ^2^ Department of Civil and Environmental Engineering University of California Irvine CA USA; ^3^ Department of Computer Science University of California Irvine CA USA; ^4^ Department of Statistics University of California Irvine USA; ^5^ Biospheric Sciences Laboratory NASA Goddard Space Flight Center Greenbelt MD USA; ^6^ Faculty of Sciences Vrije Universiteit Amsterdam Amsterdam Netherlands; ^7^ Department of Geographical Sciences University of Maryland College Park MD USA; ^8^ Global Modeling and Assimilation Office NASA Goddard Space Flight Center Greenbelt MD USA

**Keywords:** vapor pressure deficit, ocean climate indices, El Niño–Southern Oscillation (ENSO), autoregression, statistical model, fire forecasting

## Abstract

Fire emissions of gases and aerosols alter atmospheric composition and have substantial impacts on climate, ecosystem function, and human health. Warming climate and human expansion in fire‐prone landscapes exacerbate fire impacts and call for more effective management tools. Here we developed a global fire forecasting system that predicts monthly emissions using past fire data and climate variables for lead times of 1 to 6 months. Using monthly fire emissions from the Global Fire Emissions Database (GFED) as the prediction target, we fit a statistical time series model, the Autoregressive Integrated Moving Average model with eXogenous variables (ARIMAX), in over 1,300 different fire regions. Optimized parameters were then used to forecast future emissions. The forecast system took into account information about region‐specific seasonality, long‐term trends, recent fire observations, and climate drivers representing both large‐scale climate variability and local fire weather. We cross‐validated the forecast skill of the system with different combinations of predictors and forecast lead times. The reference model, which combined endogenous and exogenous predictors with a 1 month forecast lead time, explained 52% of the variability in the global fire emissions anomaly, considerably exceeding the performance of a reference model that assumed persistent emissions during the forecast period. The system also successfully resolved detailed spatial patterns of fire emissions anomalies in regions with significant fire activity. This study bridges the gap between the efforts of near‐real‐time fire forecasts and seasonal fire outlooks and represents a step toward establishing an operational global fire, smoke, and carbon cycle forecasting system.

## Key Points

- We developed a global fire emissions forecasting system with lead times of 1 to 6 months, building on a statistical ARIMAX modeling framework
- The system draws upon different data sources to optimize fire prediction in over 1,300 individual fire cohesive regions
- Our results suggested that the joint use of past fire observations within the fire season and climate data can improve S2S fire prediction

## 1 Introduction

Fire is a worldwide phenomenon that influences the structure, distribution, and function of terrestrial ecosystems (Bowman et al., 2009). It is also a major source of many trace gases, aerosols, and greenhouse gases in the atmosphere (Andreae, 2019). During the past two decades, burning from global fires released about 2.2 Pg (10^15^ g) of carbon into the atmosphere each year (van der Werf et al., 2017). Fire emissions perturb atmospheric composition and biogeochemical cycles (Yue & Unger, 2018), contribute to changes in weather and climate (Ward et al., 2012; Zhang et al., 2017), and pose risks to human health (Johnston et al., 2012).

Fire emissions worldwide exhibit considerable spatial and temporal variability (Giglio et al., 2013; van der Werf et al., 2017). Climate regulates the global distribution of fire emissions. Regions with highest levels of burning are often associated with intermediate amounts of precipitation and substantial wet‐dry seasonal cycles (Saha et al., 2019). Land surface properties, including vegetation type, topography, soil moisture, and the structure of natural and anthropogenic barriers to fire spread also play a key role in determining regional patterns of fire variability (Archibald et al., 2009; Kane et al., 2015). It is important to note that climate and land surface drivers often are not independent; climate can shape and modify surface features, and land surface properties can in turn influence local weather and regional climate.

Climate also plays a critical role in regulating the temporal variability of fires by shaping patterns of lightning ignition, fuel moisture, and fuel amount and connectivity. Many studies have documented a tight coupling between climate variables and different measures of fire activity on seasonal (e.g., Chen, Morton, et al., 2013), interannual (e.g., Abatzoglou et al., 2018; Chen et al., 2011; Jolly et al., 2015; van der Werf et al., 2008), decadal (e.g., Westerling et al., 2006), and centennial (e.g., Marlon et al., 2008; Pechony & Shindell, 2010) time scales. For example, during the positive phase of El Niño–Southern Oscillation (ENSO), burned area and fire emissions in many tropical land regions respond in a predictable way, with increases across the Maritime Continent early in the El Niño cycle followed by increases in Central and South America during the middle and later phases of these events (Chen et al., 2017; van der Werf et al., 2017).

Humans contribute to variability and long‐term trends of fires by a series of direct mechanisms regulating ignition and suppression, and indirectly through modification of fire weather and land surface properties. Humans have a long history of changing fire regimes, using fires to modify ecosystem function over time scales of millennia (Bowman et al., 2011; Marlon et al., 2008). Over the past two decades, human modification of the land surface, including the expansion of global agriculture in ancient grasslands and savannas, has contributed to a nearly 25% decline in global burned area (Andela et al., 2017). In many areas, people are responsible for more accidental or intentional fire starts than those occurring naturally as a consequence of cloud‐to‐ground lightning. This includes most areas in tropical (DeFries et al., 2010) and temperate ecosystems (Balch et al., 2017; Mollicone et al., 2006). Even in places where humans are responsible for most sources of fire ignition, climate variability can change the likelihood of fires escaping from human control and the efficacy of suppression; thus, human and climate influences on fire regimes are often difficult to disentangle.

With climate change and increasing human settlement in the wildland‐urban interface (WUI) (Radeloff et al., 2005), threats from changing fire regimes for ecosystems and human health are growing in many areas. In response to these changes, considerable effort has gone into predicting fire intensity and severity, which may help guide fire management, including fire‐fighting resource allocation, the timing and frequency of prescribed burns, and fuels management. There are multiple time scales and types of fire prediction models that are used operationally. Over the duration of a weather forecast (5–10 days), fire weather indices derived from forecast variables provide near‐real‐time warning of fire risk, including for example the fire danger forecast produced by the European Forest Fire Information System and Global Wildfire Information System (GWIS, 2019), and fire weather outlooks by the U.S. National Weather Service (SPC, 2019). After ignition, another class of models explicitly tracks the spread of individual fires using weather and fuel composition as driver variables (e.g., FARSITE and FSPro) (Finney, 2004; Finney et al., 2011). Other models predict the development of existing fires and the probability of new fires given prior and current climate and surface conditions (e.g., WIFIRE) (Altintas et al., 2015). Beyond the time scale of weather forecasts, another set of physically or statistically based methods are used to provide fire outlooks at subseasonal to seasonal (S2S) time scales (1–6 months). Examples of such operational forecast systems include National Significant Wildland Fire Potential Outlook in the United States produced by National Interagency Coordination Center (NIFC, 2019), and experimental fire season severity forecasts for the Amazon (Chen et al., 2011).

For the S2S time horizon, there is currently no fire forecast system that is global in nature, accounts for information flows arising from burning levels early in the fire season, and also has a means to seamlessly switch between different remote ocean teleconnection and local climate data streams. Such a system is needed for interpreting new fire extremes, for forecasting the response of atmospheric CO_2_ to changes in climate, and for guiding fire and air quality management in regions that lack locally tailored seasonal outlook systems.

Recent years have seen a deluge of Earth system data from satellite remote sensing and in situ measurements. The availability of these data opens up novel ways for using data‐driven methods to develop fire forecasts (Taylor et al., 2013). Past work has explored the covariance between fire regime parameters (active fire detections, fire radiative power, fire size, burned area, fire emission, fire pollutants) and various environmental controls, while use of lagged correlations between climate drivers and fire activity has provided the basis for forecasts of fire season severity (e.g., Chen et al., 2011, 2016; Fernandes et al., 2011; Jolly et al., 2015; Roads et al., 2010) or prediction of the likelihood of large fires (e.g., Coffield et al., 2019; Preisler et al., 2011). In this study we exploited a time series statistical prediction technique to create a global fire forecasting system that incorporates the memory effect of past fires, long‐term trends, as well as the influence of seasonal and interannual variability in climate. We integrated spatially resolved fire and climate data in a time series framework (Box et al., 2015). By using historical observations and an Autoregressive Integrated Moving Average model with exogenous predictors (ARIMAX) (Luceño & Peña, 2008), we derived lagged connections between the sum of fire emissions in each predefined land region (the target) and multiple explanatory variables (the predictors). Predictors considered in this study included past values and forecast errors of the target variable, as well as external predictors that represent large‐scale and local variability in climate. The optimized multivariable regression model was then used to generate forecasts of monthly fire emissions with 1 to 6 month lead times using all available data up to the date of the forecast. The goal of this paper is to develop a spatially explicit statistical model that is feasible for operational forecasts of global fire emissions, with model parameters that provide insight about regional drivers of fire activity. Our analysis also may contribute to the field of seasonal fire forecasting by isolating information originating from past fire activity within the fire season, ocean teleconnections, and regional fire weather. Our methodology for systematically evaluating the efficacy of S2S fire predictions as a function of forecast lead time and region, by means of comparison with persistence and climatology models, may be applicable to other statistical and dynamical fire modeling studies.

The outline of this manuscript is as follows. In section 2, we present the datasets used in this study. We also provide a short introduction to the ARIMAX time series modeling framework. The fire emissions and climate data we analyzed using multiple tests are described in section 3. The results from these tests helped us design appropriate geospatial regions for prediction, and select relevant predictor time series and ARIMAX hyperparameters. In section 4, we present an integrated system intended for experimental operational forecasts. In section 5, we evaluate model forecasts using in‐sample and out‐of‐sample observations, and examine how different levels of model complexity influence model performance. We then discuss model limitations and compare our model with other work in section 6. We conclude with a short summary of our approach and major findings.

## 2 Data and Model Framework

### 2.1 Data

#### 2.1.1 Fire Emissions

There are many observed or modeled data that represent different characterizations of fire occurrence and spatiotemporal variability. Here we used a geospatial dataset of global fire emissions as our main data. Based on a biogeochemical modeling system and global burned area products derived from satellite observations, the Global Fire Emissions Database (GFED) provides a continuous spatially resolved time series of fire emissions from 1997 through the present (van der Werf et al., 2017). In this study, we forecast the monthly carbon emissions of GFED Version 4 with small fires (GFED4s, downloaded from http://www.globalfiredata.org/). The original GFED4s emission data (0.25° × 0.25°, monthly resolution) were aggregated into regions with variable sizes (see section 2.2 for details) and served as our target variable for statistical model prediction.

As described in more detail below, we used GFED4s time series from 1997–2014 for model development and training, and 2015–2018 time series for prediction and model evaluation. Because production of Collection 5 MODIS burned area was halted at the end of 2016, the last 24 months of GFED4s time series during 2017–2018 were preliminary and driven by time series of active fire detections by MODIS aboard Aqua and Terra (MCD14ML, Collection 6). This preliminary product was created by regressing MODIS active fires with GFED4s emissions time series in each grid cell during 2003–2016. The emissions estimates from this approach were found to be within 2% of the standard GFED4s emissions for continental and annual scales, indicating that the active fire observations are able to capture most of the variability in fire emissions on seasonal and interannual time scales. We included the preliminary GFED4s time series in our evaluation to allow us to examine the model performance during the El Niño and La Niña periods.

#### 2.1.2 Ocean Climate Indices

**Figure 1:**
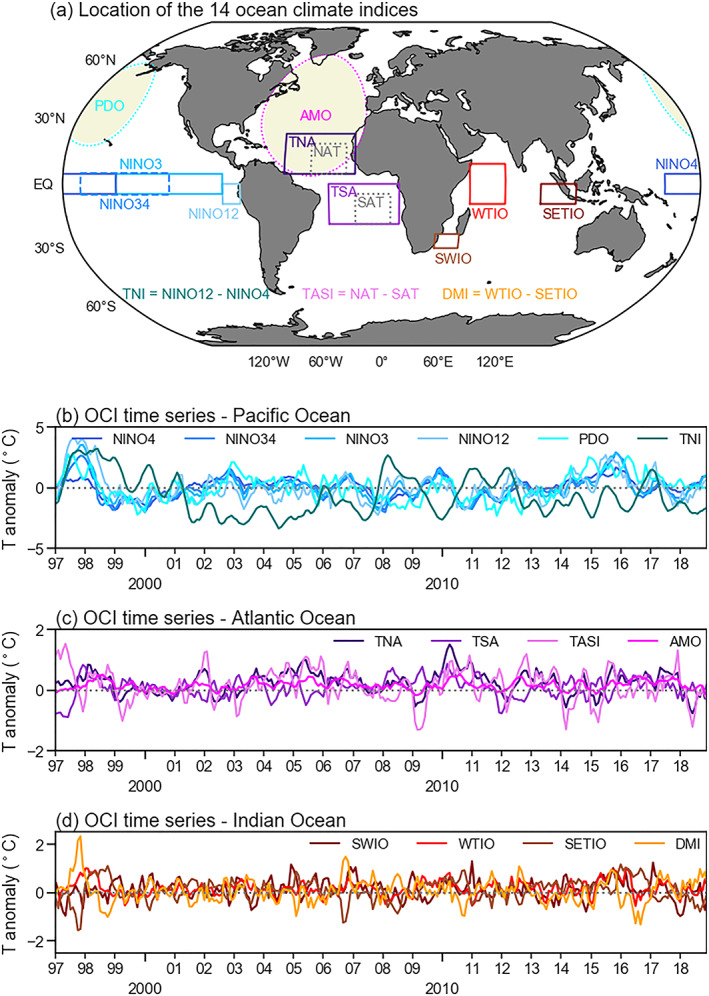
Location of the 14 ocean climate index (OCI) regions used in this study (a). Note that the NAT (North Atlantic Tropical SST index) and SAT (South Atlantic Tropical SST index) regions were used only for defining TASI (Tropical Atlantic SST index) but not used as predictors in the ARIMAX model. (b, c, and d) Time series of the OCIs in different oceans during 1997–2018. Please refer to Table S1 for more detailed information on these OCIs.

Ocean climate indices (OCIs), representing the departure from the mean state of sea surface temperatures (SSTs) averaged over different ocean regions, are often used to characterize major patterns of large‐scale climate variability. By using multiple observational sources, the U.S. National Oceanic and Atmospheric Administration (NOAA) has developed different OCIs with regular updates, often on a weekly or monthly basis. We downloaded 14 OCIs from the NOAA State of the Ocean website (http://stateoftheocean.osmc.noaa.gov/sur/) and converted the data to monthly mean values, when necessary. Six of these OCIs represent the ocean status for different regions in the Pacific, four for the Atlantic, and four for the Indian Ocean. The location and time series of OCIs for the study period in different oceans are shown in Figure 1 and supporting information Table S1.

#### 2.1.3 Vapor Pressure Deficit

Recent studies have suggested that vapor pressure deficit (VPD), a meteorological parameter representing the difference between the amount of moisture in the air and how much moisture the air can hold when it is saturated, is closely linked with fuel moisture and thus variability of fire activity in some regions (Abatzoglou & Williams, 2016; Coffield et al., 2019; Seager et al., 2015; Sedano & Randerson, 2014; Williams et al., 2019). We extracted monthly 2 m air temperature (*T*, in Celsius), 2 m specific humidity (*Q*_*v*_, in g/g) and surface pressure (*P*_*s*_, in hPa) values from the Modern‐Era Retrospective analysis for Research and Applications, Version 2 (MERRA‐2) data (instM_2d_asm_Nx as described in Bosilovich et al. (2015)). MERRA‐2 meteorological data were derived from an assimilation system that optimally combines physical theory with multiple sources of satellite observations (Gelaro et al., 2017). The original MERRA‐2 *T*, *Q*_*v*_, and *P*_*s*_ data were downloaded from NASA Goddard Earth Sciences Data and Information Services Center (https://disc.gsfc.nasa.gov/) and regridded to a 1° × 1° spatial resolution. We then derived saturation vapor pressure (*e*_*s*_, in hPa) from *T* using the Clausius‐Clapeyron equation *e*_*s*_ = 6.112*e*^(17.67 × *T*)/(*T*+243.5)^ (Bolton, 1980), and the actual vapor pressure (*e*_*a*_, in hPa) from *P*_*s*_ and *Q*_*v*_ ( $e_{a}=Q_{v}\times \frac{P_{s}}{\mathrm{MW}}$). *MW* (≈ 0.622) is the molecular weight ratio of water vapor to dry air. The difference between the saturated and actual vapor pressure in each 1° × 1° grid cell was recorded as the mean VPD value.

#### 2.1.4 Country Mask

To help define fire forecasting regions, we used a map of individual country locations from Natural Earth (https://www.naturalearthdata.com/). The mask was regridded to a 1° × 1° resolution, corresponding to the highest spatial resolution of our regional fire forecasts.

### 2.2 Defining Fire Cohesive Regions for Forecasts

Fire emissions variability, as well as its dependence on climate, ecosystem, and human drivers, is highly heterogeneous across different regions and continents. Therefore, a universal statistical model that can reproduce all spatiotemporal variations in fire emissions is currently infeasible and impractical. An alternative approach is to divide the global land area into cohesive regions and derive region‐specific models with regionally tuned parameters. However, selecting the optimal set of regions can be challenging: small grid cells may suffer from insufficient data as a consequence of infrequent fires, whereas large grid cells may lead to blending of different influences from land surface, climate, and human drivers. In this study we examined historical monthly GFED4s data (for the period of 1997–2014) to define fire cohesive regions (FCRs) suitable for fire emissions forecasting. These FCRs leverage the spatiotemporal structure of recent fire activity for near‐term forecasts, including the contemporary distribution of human and natural ignitions, landscape fragmentation, and fuels.

To derive the FCRs, we first defined regularly shaped grid cells on a global map using spatial resolutions of 1° × 1°, 2° × 2°, 4° × 4°, 8° × 8°, respectively. The grids at different resolution levels were perfectly aligned so that an upper‐level grid cell contained exactly four lower‐level grid cells (except for the northernmost 8° × 8° grid cells, which contained only two 4° × 4° subcells).

**Figure 2:**
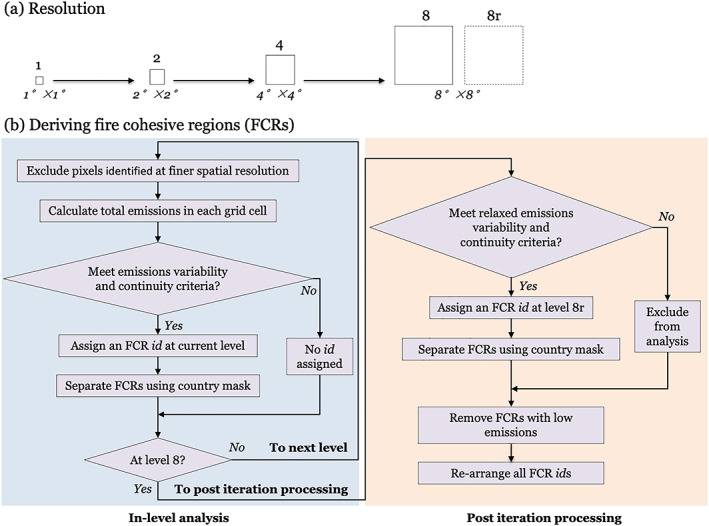
(a) Fire cohesive regions (FCRs) were defined by progressively iterating from the finest spatial resolution (1° × 1°) up through to the coarsest spatial resolution (8° × 8°). (b) A flowchart of steps required to develop the FCRs for fire emissions forecasts. At each level, total fire emissions in grid cells for a candidate FCR were tested using multiple criteria (in‐level analysis). Each grid cell or set of grid cells satisfying all of the criteria was considered as an individual FCR. After iterating up through the 8° × 8° level, additional postiteration analyses were performed to develop global FCRs for remaining grid cells that satisfied the relaxed emissions variability and continuity criteria.

We started by examining the magnitude and temporal variability of total fire emissions within each 1° × 1° grid cell (Figure 2). We considered the following three criteria to determine whether a grid cell (at a 1° × 1° resolution) should be defined as an individual FCR. First, we required that fire emissions exceeded a minimum threshold within each region. We set this threshold so that total fire emissions had to be equal to or be above the cutoff value for the top 2% of land grid cells (1° × 1°) worldwide, that is, 1.33 Tg C yr^−1^. Second, recognizing that a linear regression system may not be suitable for data with a substantial proportion of zeros, we required that nonzero emissions occurred in at least 90% of the years in the time series (i.e., burning had to occur in at least 17 out of the 18 yr of the record during the training interval). Third, we attempted to avoid cases in which the temporal variability was determined by only a few (usually anomalously large) data samples. To meet this criterion, we required that the coefficient of variation (the ratio of standard deviation to the mean) of the fire emission time series was less than 2. We considered each 1° × 1° grid cell as an FCR when all three criteria were met. We then assigned an FCR identification to each of these grid cells.

We repeated the process iteratively at levels of coarser spatial resolution, excluding all of the areas (and emissions) included in FCRs defined at the finer resolution level. At a coarser spatial resolution in which total fire emissions were aggregated over a larger area, the criteria described above generally became easier to satisfy. Recognizing large country to country differences in resources available for fire management, if the area within an FCR crossed over country borders, we divided this FCR into multiple FCRs while maintaining the assigned resolution level. Finally, we assigned individual identification numbers for each FCR defined at each resolution level.

For regions at an 8° × 8° spatial resolution that did not satisfy our condition but had mean emissions greater than 0.12 Tg C yr^−1^, we still assigned FCR ids for them and classified them as an additional Resolution Level *8r*. The final FCRs were composed of 1 or more 1° × 1° grid cells, depending on the resolution level at which they were defined. Note the number of 1° × 1° grid cells (representing the area) in some FCRs was smaller than that defined at the corresponding resolution level (e.g., 16 for Level 4 FCRs; see Figure 2), due to the exclusion of grid cells that have already contained in other FCRs at finer resolution level, and the separation of FCRs along country borders.

**Figure 3:**
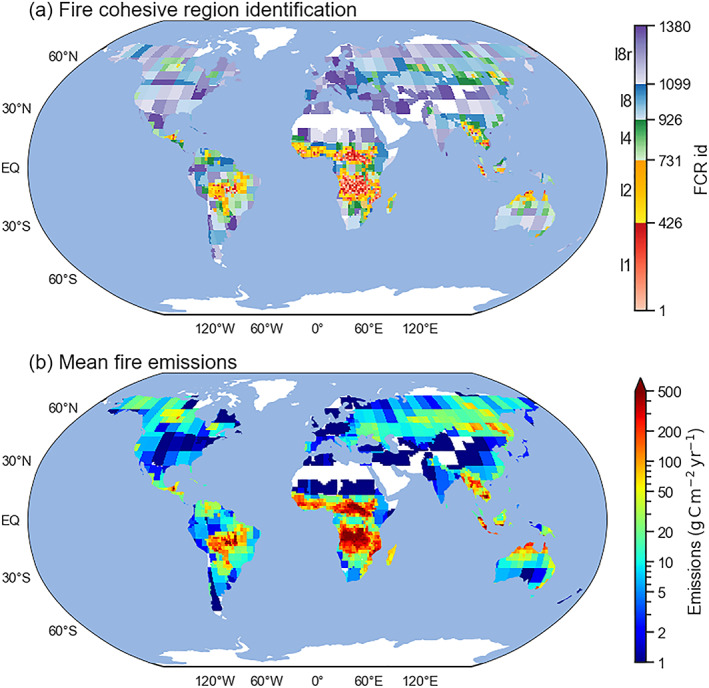
Global maps of (a) fire cohesive region (FCR) identification number and (b) mean annual fire emission density (g C m^−2^ yr^−1^, averaged over 1997–2014) in each FCR. White areas are regions with very small fire emissions (mean emissions within each region smaller than 0.12 Tg C yr^−1^).

Following all steps mentioned above, we classified global land fire regions (21,524 1° × 1° grid cells) into 1,380 FCRs (Figure 3), with 426, 305, 195, 173, and 281 in Levels 1, 2, 4, 8, and 8r, respectively. The remaining areas that did not have fire activity meeting these criteria (mostly in deserts and polar regions) accounted for 23.1% of the global land surface and less than 1.5% of global fire emissions. These areas were assumed to be fire‐free, and were excluded from subsequent forecasts.

Overall, areas with high fire emission densities had corresponding low‐level (high spatial resolution) FCRs. In contrast, areas with lower emission densities and more infrequent fire activity were aggregated into larger FCRs for the purpose of statistical fire forecasting. By this classification, the sum of fire emissions in each FCR level was comparable in magnitude and variability (Figure S1) except for Level *8r*. Level 1 FCRs, which were mostly located in tropical areas, occupied less than 5% of the global land region area, yet accounted for about half of global fire emissions (Table 1).

**Table 1:** Fire Emission Statistics in Different Fire Cohesive Region (FCR) Resolution Levels

Level	ID range	Number of regions	Percent of total number of regions (%)	Area (10^8^ km^2^)	Percent of total land area (%)	Fire emissions (Pg C yr^−1^)	Percent of total emissions (%)
1	1–426	426	30.9	0.05	3.5	0.97	45.0
2	427–731	305	22.1	0.10	6.9	0.56	25.7
4	732–926	195	14.1	0.17	11.2	0.27	12.3
8	927–1,099	173	12.5	0.33	22.3	0.22	10.0
8r	1,100–1,380	281	20.4	0.49	33.1	0.12	5.6
Low‐fire region				0.34	23.1	0.03	1.4
Total				1.48		2.17	

*Note*. Fire emissions were based on the Global Fire Emissions Database Version 4s (GFED4s) data for 1997–2014.

### 2.3 The ARIMAX Modeling Framework

We selected the ARIMAX model as our prediction model, because it provides a flexible framework for time series analysis while parameters are still sufficiently constrained to allow their interpretation in the light of large‐scale patterns of vegetation and climate. The ARIMAX model includes several components that have the capacity to capture important temporal structure in time series data. *AR* stands for “autoregression,” and captures the dependence of an observation on some number of lagged observations. *I* stands for “integration,” which can make the time series stationary by applying different orders of differencing. *MA* stands for “moving average,” and accounts for the dependency of an observation on a residual error from a moving average model applied to lagged observations. ARIMAX is an extension of the simpler ARIMA model (Piwowar & Ledrew, 2002). The inclusion of *X* component enables the model to use not only lagged observations, but also external variables as predictors. ARIMA model and its variations, including ARMA, ARIMAX, SARIMA (seasonal ARIMA) and SARIMAX, have been historically used for analyzing and forecasting Earth science data, such as drought (Jalalkamali et al., 2015), arctic sea ice (Piwowar & Ledrew, 2002), vegetation greenness (Fernandez‐Manso et al., 2011; Ji & Peters, 2004), burned area (Riano et al., 2007), and aerosol optical depth (Abish & Mohanakumar, 2013).

In this study, we applied the ARIMAX scheme (Durbin & Koopman, 2012) in each FCR to create a global fire emission forecast system. A mathematical form of the ARIMAX model used for each FCR is shown in Equation 1:

(1) $$y_{t}^{\prime d}=c+\sum_{i=1}^{p}\alpha_{i}y_{t-i}^{\prime d}+\sum_{j=1}^{q}\beta_{j}\epsilon_{t-j}+\sum_{k=1}^{n}\gamma_{k}x_{k}+\epsilon_{t}$$

in which *y*_*t*_ is our target variable of monthly emissions at time *t*, *ϵ*_*t*_ is the modeling error, and *x*_*k*_ represents exogenous predictors. *c*, *α*_*i*_, *β*_*j*_, and *γ*_*k*_ are regression parameters optimized from the training data. The value of *y*_*t*_ is expressed as a combination of linear regression components, as shown on the right‐hand side of Equation 1 (from left to right, in order, the intercept, *AR*, *MA*, *X*, and the error). AR and MA components include linear terms that are derived from one or multiple (up to *p* and *q*, respectively) prior observations and errors. The raw observations (*y*_*t*_) are first transformed to $y_{t}^{\prime d}$ by differencing (the inverse of integration, representing the *I* component of ARIMAX) for an order of *d*, which makes the time series stationary and facilitates the linear model fitting in practice. For example, in the case of first order differencing (*d* = 1), the changes between consecutive observations are used in the transformation ( $y_{t}^{\prime 1}=y_{t}-y_{t-1}$). The three hyperparameters *p*, *d*, and *q*, corresponding to the orders of the *AR*, *I*, and *MA* components, need to be carefully selected in order to produce optimal model predictions (Table 2 and section 4.3).

**Table 2:** Components and Parameters of the ARIMAX Model Used in This Study

Component	Name	Parameter	Parameter optimization range
Autoregression	AR	*p*	0–2
Integration	I	*d*	0–1
Moving average	MA	*q*	0–2
Ocean climate index	X (OCI)	OCI, lag	1–11 months
Vapor pressure deficit	X (VPD)	lag	1–11 months

In the ARIMAX model, one or more (up to *n*) exogenous predictors (*x*) with the same dimension as the data (*y*) can be used. In this study we used a local meteorological parameter, VPD, and one of the 14 OCIs as exogenous predictors for fire emissions in each FCR. For each prediction, we selected the optimal OCI type as well as optimal lag time for VPD and OCI according to cross correlation analyses described in section 4.2. The hyperparameters (*p*, *d*, and *q*) and all the coefficients in the regression model (*c*, *α*_*i*_**,***β*_*j*_**,***γ*_*k*_ in Equation 1) were allowed to be FCR‐specific.

We implemented the ARIMAX model fitting and prediction using the *SARIMAX* module in the *StatsModels* python package (https://www.statsmodels.org).

## 3 Data Analyses Over the Model Development Period (1997–2014)

The ARIMAX model is based on a range of assumptions about the underlying data and also has multiple variations suitable for different applications. In implementing an ARIMAX model for fire forecasting, it is necessary to identify the fire emissions time series patterns, and to explore the optimal parameterization of each of the AR, I, MA, and X components. Here we followed a standard approach (Box et al., 2015; Hyndman & Athanasopoulos, 2018) and used statistical tests for guiding the selection of ARIMAX model data and parameters (Table 2). These tests were performed on fire emission data in different FCRs during a recent historical period (1997–2014) that was used for model development in this study. In particular, we tested the seasonality (section 3.1), stationarity (section 3.2), and temporal autocorrelation (section 3.3) of the target data of fire emissions, which can be used to determine the range of feasible *p*, *d*, and *q* values, and any required data transformations. We also explored several exogenous variables to evaluate their predictive power (section 3.4). Results from these tests therefore provided the basis for selecting ARIMAX model parameters and establishing an integrated forecasting system (section 4).

### 3.1 Seasonality of the Emission Data

Month‐to‐month variations in fire emissions showed distinctive seasonal cycles in many fire regions (Figure S1), which led to substantial spatial differences in both the peak burning month and the length of the burning season (Figure S2). These seasonal patterns were often associated with the timing of dry and wet seasons, the growth of herbaceous biomass and fuels, and human activity.

Our analysis of the GFED emissions indicated that the annual fire cycle did not change significantly from year to year in most FCRs. Therefore, we first removed this annual cycle from the original data, and then applied the ARIMAX model to the time series of residual fire emission anomalies (described below in section 4.1). An alternative approach is to add a seasonal component to the ARIMAX model (i.e., the SARIMAX model) to automatically account for the seasonality of a time series data. The seasonal component in SARIMAX has the same structure as the nonseasonal component, which includes autoregression, integration, and moving average terms. The only difference is that the value from the previous season is used as the prior observation. In our case, with 12 intervals (months) in a full cycle (year), fire emissions in the same month of the previous year would be used as a predictor. However, we found the addition of a seasonal component in the SARIMAX model did not elevate the forecast performance enough to offset the increasing computational cost and likelihood of over fitting (data not shown).

### 3.2 Stationarity of the Emission Data

In time series analysis, stationary data are often necessary, whereby the mean, variance and autocorrelation structure of the time series does not change over time. Real‐world data such as fire emissions used in this study, however, are often nonstationary. For example, Andela et al. (2017) found a significant decreasing long‐term trend in satellite‐derived burned area over many savanna and grassland regions. If a time series is nonstationary, preprocessing steps can be taken to convert it to a stationary time series. In the ARIMAX model, it is possible to set a nonzero order (*d*) for the integration part (*I*) to accomplish this transformation. Here we checked the stationarity of fire emission time series in each FCR by calculating the long‐term trend (section 3.2.1) and performing Kwiatkowski‐Phillips‐Schmidt‐Shin (KPSS) tests (Kwiatkowski et al., 1992) (section 3.2.2).

#### 3.2.1 Trend Analysis

From 1997–2014, fire emissions in equatorial Asia, Central America, and northeastern Asia regions showed significant decreasing trends (Figure S3a). These trends may be partly attributable to land use change, including the conversion of savannas to croplands, as well as variability in climate (e.g., there was a strong El Niño at the beginning of the time series in 1997 and 1998). Strong positive trends occurred in northern Canada and northern Siberia, and were at least partly attributable to climate warming in high northern latitude regions (Gillett et al., 2004; Veraverbeke et al., 2017).

#### 3.2.2 Trend Stationary Test

KPSS tests are traditionally used to test a null hypothesis that an observable time series is stationary around a constant (c, the mean value) or a deterministic trend (ct). We performed KPSS tests on the GFED fire emissions time series during 1997–2014 (Figures S3b and S3c) to provide complementary information on the order range of differencing in the ARIMAX model (actual values of *d* in our practical forecasting were determined by local optimization, as detailed in section 4.3). These analyses showed fire emissions in about 10% of the total FCRs were nonstationary (*p* < 0.05) around a constant, indicating that a nonzero order of differencing in the ARIMAX model would be the most suitable setting for this hyperparameter. Most of the nonstationarity in this set was associated with a long‐term trend, and only 2.5% of the FCRs were nonstationary around a trend (*p* < 0.05). These results suggested it is sufficient to set a zero or one order of differencing (*d* value in the ARIMAX model) in most FCRs.

### 3.3 Autocorrelation of the Emission Data

The strength of a relationship between an observation in a time series and observations at prior time steps can be evaluated using the autocorrelation function (ACF) and the partial autocorrelation function (PACF). The ACF pattern indicates how many MA terms are likely needed to be used (i.e., the *q* value in Equation 1). The pattern of PACF, which is the amount of correlation that is not explained by correlations at all lower‐order lags, is useful for determining the number of AR terms needed (i.e., the *p* value).

Figure S4 presents the ACF and PACF of monthly GFED fire emissions (after removing the seasonality) as a function of lag time. At short lags, the ACFs were often large positive values. A spatial map of ACFs at a 1 month lag (Figure S4c) provided evidence for very strong autocorrelation in Australia and in boreal forest regions. The ACF decayed at different rates in different FCRs. It turned negative in some regions when the lag was over 2 months. Unlike the ACF, which decayed slowly, the PACF often displayed a sharp cutoff at a 1 month lag, suggesting the number of AR terms (*p*) is expected to be around 1 for most regions.

The spatial and temporal variations in autocorrelations suggested that the role of autoregression in fire emission forecasting was not the same in different regions or for different forecast lead times. For example, emissions anomalies at the beginning of a fire season may correlate either positively or negatively with emissions anomalies at the end of the fire season, depending on the ecosystem type and continental region. Strong positive correlations between early and late fire season fire emissions were found in most fire regions (Figure S5), implying that meteorological or human controls had a certain level of consistency throughout the fire season. In some regions, however, including mesic African savannas north and south of the equator, fire emissions early in the fire season were often negatively correlated with emissions near the end of the fire season (shown in blue in Figure S5). This negative association may be caused by contrasting climate anomalies during early and late phases of the fire season. Another likely mechanism is that higher than normal fires in the early part of the season deplete fuels (amount or continuity), thereby limiting late‐season fires. These spatially variant strong correlations, positive or negative, highlight the importance of using a region‐specific autoregression term in a global fire forecasting system.

### 3.4 Correlation Between Fire Emissions and Large‐Scale Climate Indices

Previous studies (e.g., Chen et al., 2011, 2016, 2017; Fernandes et al., 2011; Pan et al., 2018) have shown strong links between the fire season severity in some regions and SST‐derived OCIs. Here we systematically calculated time‐lagged (0–11 month) cross correlations between fire emission anomalies in each FCR and different OCIs. These results, which are shown for NINO3.4 in Figure S6a and for AMO in Figure S6b, confirmed the influence of large‐scale ocean‐atmosphere climate interactions on the temporal variability in fire emissions in many regions and that the sensitivity of fire emissions to individual OCIs can be very different. For a particular FCR‐OCI pair, the correlation also depended on the time of lag between emissions and the OCI. We used these lagged correlations to identify the optimal OCI time series (and lag time) in the design of our global forecasting system (see section 4 for details).

### 3.5 Correlation Between Fire Emissions and Local Meteorology

Local meteorological variables have also been used in statistical fire forecasts (Shen et al., 2019) because they constrain local fuel amount and fuel moisture which are needed for fire occurrence. While many local meteorological parameters (such as temperature, precipitation, and wind speed) provide information for fire seasonal outlook, here we used a simple indicator of surface moisture stress, VPD, as an exogenous predictor for fire emissions together with the optimal OCI time series. Similar to OCI, the influence of VPD on fire emissions depended on the lag time (Figure S6c). In many regions, high positive correlations occurred at short lag times. Low correlations were more dominant for longer lag times.

## 4 Establishing an Integrated Forecast System

**Figure 4:**
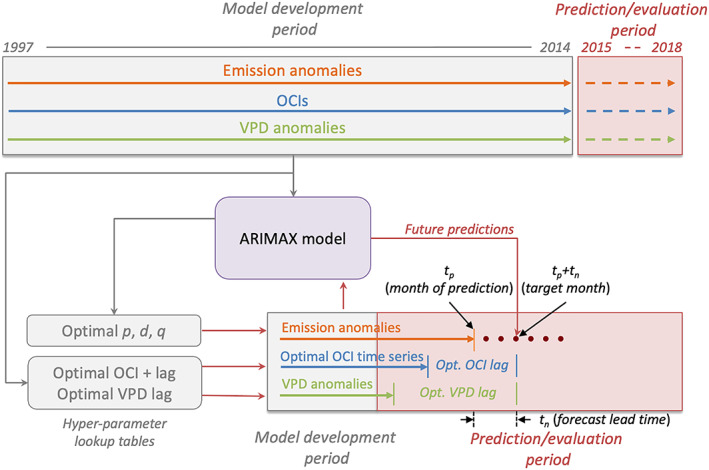
Flowchart of the experimental fire emissions forecasting system developed in this study. Using data in the model development period, we first derived look‐up tables of hyperparameters, including the optimal *p*, *d*, and *q* values (with ARIMAX model used), the optimal ocean climate index (OCI) type, and the optimal lag times for OCI and vapor pressure deficit (VPD) exogeneous climate variables within each fire cohesive region (FCR). Then for each forecast interval, we derived a series of future predictions (lead times of 1–6 months) based on the corresponding hyperparameters extracted from the look‐up tables and the time series of fire emissions and climate data available up through the month of the forecast. The model development period and the process for deriving hyperparameters are shown in gray, while the out‐of‐sample prediction period and forecast methodology are shown in red.

To create a forecast system of fire emissions using the ARIMAX model, we first prepared the time series of fire emissions (section 4.1) and all potential climate predictors (section 4.2) for each FCR. We split the full dataset of 1997–2018 into a period for model development (1997–2014) and a period for prediction and model evaluation (2015–2018). By using data and predictors in the development period, we fit the ARIMAX model and derived a look‐up table of optimal hyperparameters (section 4.3). Then for each month in the prediction period, we used the ARIMAX model to make a series of experimental forecasts with 1 to 6 month lead times, using optimized hyperparameters from the development period and all available data (emissions, OCIs, VPD) up to the date of prediction (section 4.4). Finally, we tested the performance of the ARIMAX model forecast by turning off different components of the model, and by comparing it to that from other conventional forecasting models (section 4.5). A flowchart of the operational forecast is given in Figure 4.

### 4.1 Standardization and Transformation of Fire Emission Data

We first normalized the monthly fire emission data (*y*_*t*_) by removing the climatological monthly mean ( ${\bar{y}}_{m\left(t\right)}$, derived from the model development period) and then dividing by the interannual standard deviation (*σ*_*m*(*t*)_) for each calendar month (*m*). In order to account for non‐Gaussian distributions of fire emissions and avoid extreme high values, we also used hyperbolic tangent (*tanh*) as a mapping function to transform the normalized values. After normalizing and transforming (Equation 2), the fire emission anomaly data ( ${\hat{y}}_{t}$) were restricted to the range of [−2, 2] (Figure S7).

(2) $${\hat{y}}_{t}=2\times \tanh\left(\frac{y_{t}-{\bar{y}}_{m\left(t\right)}}{2\times \sigma_{m\left(t\right)}}\right)$$

**Figure 5:**
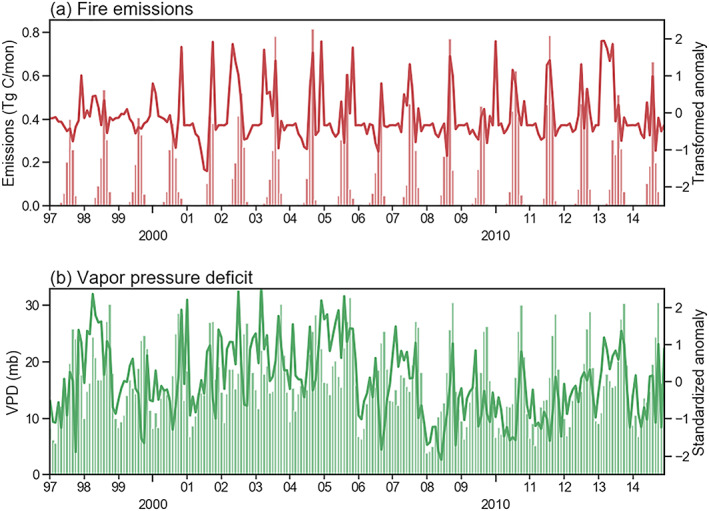
Example time series data used for a fire emissions forecast for a fire cohesive region (FCR) in southern Africa (FCR *id* = 321, centered at [19.5E, 16.5S]). (a) Monthly fire emissions (bars) and the transformed emissions anomaly time series (line). (b) Monthly vapor pressure deficit (VPD) (bars) and standardized VPD anomaly time series (line).

Figure 5a shows the original emissions and processed anomaly time series of fire emissions in a sample FCR during the model development period.

Similarly, we calculated the climatological mean and standard deviation of VPD values for each month in each FCR. By removing the mean and dividing by the standard deviation, we transformed the original VPD to a time series of standardized VPD (the *tanh* transformation was not applied since VPD's marginal distribution is close to being normal while fire emission's marginal distribution is right skewed). An example VPD time series during the development period is shown in Figure 5b.

### 4.2 Optimal Type and Lead Time of Exogenous Predictors

**Figure 6:**
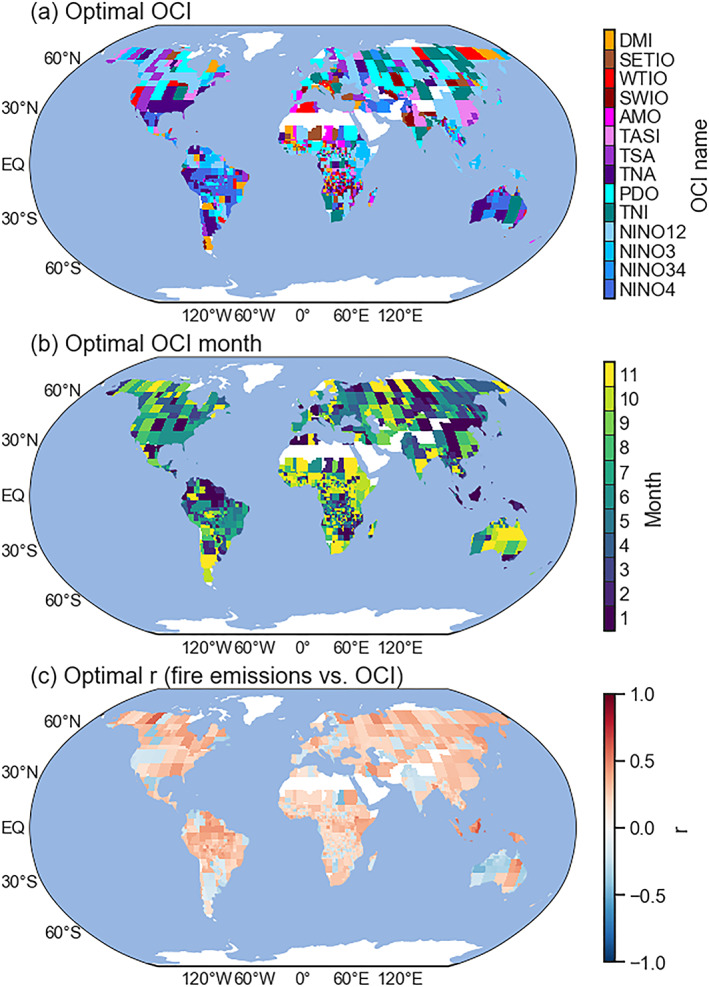
Global maps of (a) optimal Ocean Climate Index (OCI) type, (b) optimal lag time (in months), and (c) the associated correlation coefficient (*r*) between fire emissions and OCI at the optimal lag time in Panel b. All panels correspond to the case of a 1 month forecast lead time.

We considered two exogenous predictors (*n* = 2 in Equation 1) in the ARIMAX model: a time‐lagged optimal OCI time series and a time‐lagged VPD time series. Based on the lagged cross correlations (Figure S6) between historical fire emission anomaly data and 14 OCIs operationally produced by NOAA for each FCR (Figure 1), we derived the OCI type and lag time pair that had the largest correlation coefficient (in absolute value) with emissions for a given forecast lead time (Figure S8). The forecast lead, which is defined as the difference between the date of prediction and the date of target, determined the minimum lag we were able to use in the operational forecast. Figure 6 presents a global map of optimal OCI type, OCI lag, and the optimal correlation for the case of a 1 month forecast lead. We recorded all optimal OCIs and associated optimal lags (as a function of forecast lead time) for each FCR in a look‐up table for later use in the forecasting system.

**Figure 7:**
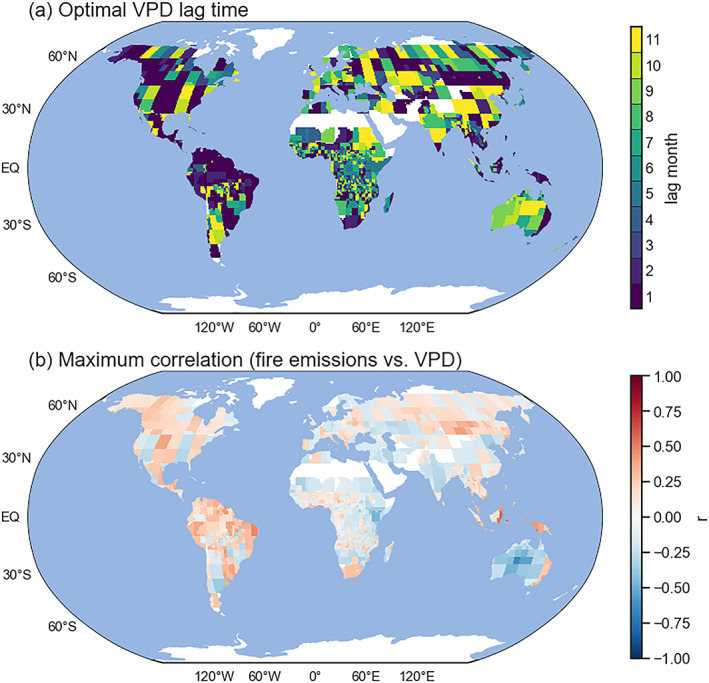
Global maps of (a) optimal lag time (in months), and (b) the associated correlation coefficient (*r*) between fire emissions and vapor pressure deficit (VPD) at the optimal lag time shown in Panel a. Both panels correspond to the case of 1 month forecast lead time.

Similarly, by using lagged correlations between fire emissions and VPD (Figure S6c), we created a look‐up table of the optimal lag for the emissions−VPD relationship for each FCR and forecast lead time (Figures 7 and S9). We assumed these cross correlation‐derived parameters provided the best exogenous predictors for the ARIMAX forecast.

### 4.3 Orders of Autoregression, Differencing, and Moving Average

**Figure 8:**
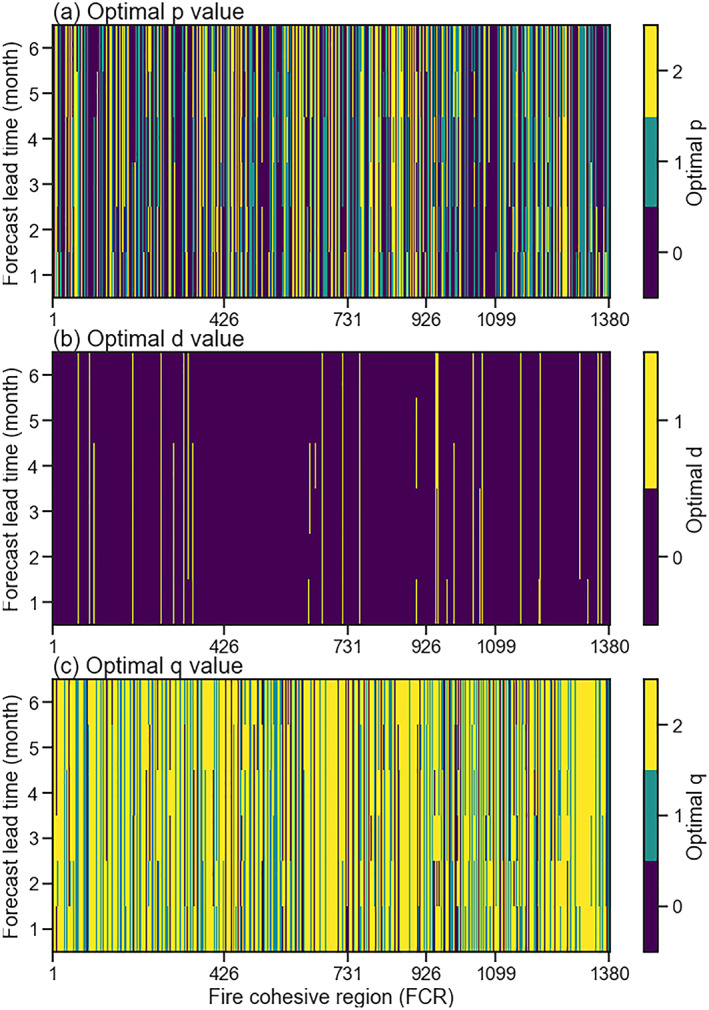
The optimal (a) *p*, (b) *d*, and (c) *q* values for the *OCIVPDAR* model (ARIMAX with OCI and VPD exogeneous climate variables; see Table 3) at different forecast lead times (1–6 months).

The *p*, *d*, and *q* values in Equation 1, representing the orders of autoregression (AR), differencing (*I*), and moving average (MA) hyperparameters, are needed for the ARIMAX model. Here, we looped over all combinations of *p*, *d*, and *q* (shown in Table 2) to find the optimal values for ARIMAX in each FCR and each forecast lead time. The range of potential values for *p*, *d*, and *q* were determined from previous analyses of fire emissions seasonality, stationarity, and autocorrelation (see section 3) as well as computational feasibility. For *p* and *q*, we used a range of 0 to 2 months. We also set the option of *d* parameter to be either 0 or 1. The optimal configurations were determined using the Akaike's Information Criterion (AIC) (Akaike, 1974) for each potential combination. AIC is an estimator of the relative quality of statistical models for a given dataset, which penalize the goodness of fit with the number of parameters (complexity) of the model. Lower AIC values generally suggest a better model fit. The optimizations for *p*, *d*, and *q* were based on data from the model development period (1997–2014), and the resulting hyperparameters were saved in a look‐up table (Figure 8) for use in our global forecasting system.

### 4.4 Model Forecast Settings

For each month (date of forecast) in the prediction period (2015–2018) (Figure 4), we created a set of fire emission predictions 1 to 6 months into the future (*t*_lead_) in each FCR. The model hyperparameters in each FCR, including the optimized OCI type, the optimized lag times for OCI and VPD, and the optimal *p*, *d*, and *q* values, were extracted from the precalculated look‐up tables described above. All of these hyperparameters varied as a function of the forecast lead time. We shifted the OCI and VPD time series by the optimal lag extracted from the look‐up table, and used them as the exogenous predictors in the ARIMAX forecast. Since the optimal lags were no shorter than the forecast lead, this approach ensured the availability of these predictors at the month of prediction (assuming the observed data were available for the month of the forecast).

**Figure 9:**
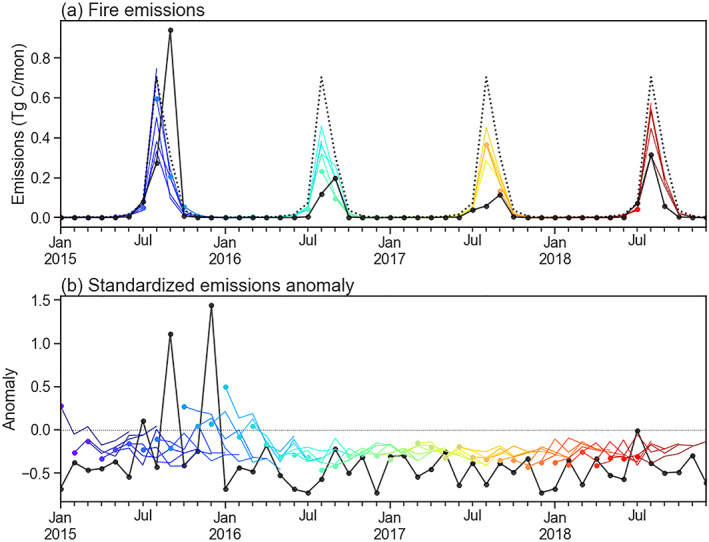
Data and operational forecasts of (a) fire emission and (b) emissions anomalies (standardized using mean emissions) in a Level 4 sample fire cohesive region (FCR) in Indonesia (111°E, 0.5°S) during the validation period (2015–2018). Emission data were from the Version 4s of Global Fire Emissions Database (GFED4s). Different colors indicate predictions at different months. Six consecutive forecasts were made at each month. The first predictions (i.e., lag time = 1 month) are shown in filled dots. Dashed lines represent the climatology, and the solid black line presents the GFED4s data.

For each prediction, the model operated on all available data in the prediction period up to the date of prediction, including data from the model development period. At the time of each prediction in each FCR, the *α*, *β*, and *γ* parameters along with the intercept (*c*) in Equation 1 were optimized using the full length of the time series available at the time of prediction. After obtaining the predictions from the forecast, we performed a reverse mapping of Equation 2 to transform the normalized anomalies back into actual fire emission data, as shown for a single FCR in Figure 9.

### 4.5 Forecast Model Experiments

In addition to the reference model (*OCIVPDAR*) where both OCI and VPD predictors were combined with autoregression, integration, and moving average (ARIMA) components of the model, we also tested several other variations to separate the impacts of different model components on overall forecast performance (Table 3). *OCIAR* and *VPDAR* models were similar to *OCIVPDAR* model, but used only one exogenous climate predictor. In the *ARonly* model, we removed the *X* component in ARIMAX, relying solely on the autoregression, integration, and moving average (*ARIMA*) of the target data for the purpose of forecasting. In contrast, for the *Xonly* model, we excluded the *ARIMA* part by assigning zeros to *p*, *d*, and *q*. For comparison, we also derived two sets of predictions using simple models: the *Clim* model used the monthly means from the model development period for the prediction (i.e., all predictions have zero anomaly values), while the *Persistence* model assumed that the anomaly of the target month was equal to the anomaly at the time of prediction.

**Table 3:** A List of Forecast Models Used in This Study

Name	Description	ARIMA	OCI	VPD
*OCIVPDAR*	ARIMAX with OCI and VPD	optimal *pdq*	yes	yes
*OCIAR*	ARIMAX with OCI	optimal *pdq*	yes	no
*VPDAR*	ARIMAX with VPD	optimal *pdq*	no	yes
*ARonly*	ARIMA (without OCI or VPD exogeneous climate variables)	optimal *pdq*	no	no
*Xonly*	Linear regression with OCI and VPD	*pdq* = 0	yes	yes
*Clim*	Prediction from a fire emissions monthly climatology	no	no	no
*Persistence*	Prediction using the observed emissions anomaly at the time of the forecast; does not change with forecast lead time	no	no	no

## 5 Model Evaluation

### 5.1 In‐Sample Validation

We first examined how well modeled fire emissions compared with data during the in‐sample (model development) period. For this comparison, we used the predicted time series from the *OCIVPDAR* model with a 1 month of forecast lead time. The global sums of monthly fire emissions from observation and model are shown in Figure S10.

Overall, our reference ARIMAX model (*OCIVPDAR*) captured about 65% of the temporal variability in global fire emissions anomalies for the in‐sample period. The model bias was considerably smaller than that from the *Clim* model. However, the ARIMAX model still underestimated emissions in some extremely high fire seasons (e.g., 1997), which also likely contributed to the low bias in global fire emissions.

### 5.2 Cross Validation

While in‐sample comparisons may provide some insight about how the model system reproduces the target variable, a more meaningful and rigorous test is to evaluate performance using out‐of‐sample data that were not used to train the model. Here we evaluated our model by using an ongoing, adaptive approach that is similar to the Walk forward analysis (Pardo, 2011). As introduced above, we made a series of forecasts for each month during the prediction period. For each monthly time step during the prediction interval, all data at and before the forecast were considered as the training set, and data during the prediction interval (1–6 months after the date of forecast) were considered as the test set. This process was repeated at subsequent time steps. The model predictions in the test set eventually spanned all of the months in the prediction period (2015–2018) shown in Figure 4. We recorded all of the model predicted emissions and compared them with the observed time series (GFED4s fire emissions) for validation. Specifically, we used correlation coefficients and root‐mean‐square error (RMSE) to investigate performance of the various models at the global scale (section 5.2.1) and per FCR (section 5.2.2). We also examined model performance for several key fire regions where fires contribute disproportionally to the global carbon cycle (section 5.2.3).

#### 5.2.1 Comparison With Global Fire Emissions Time Series

**Figure 10:**
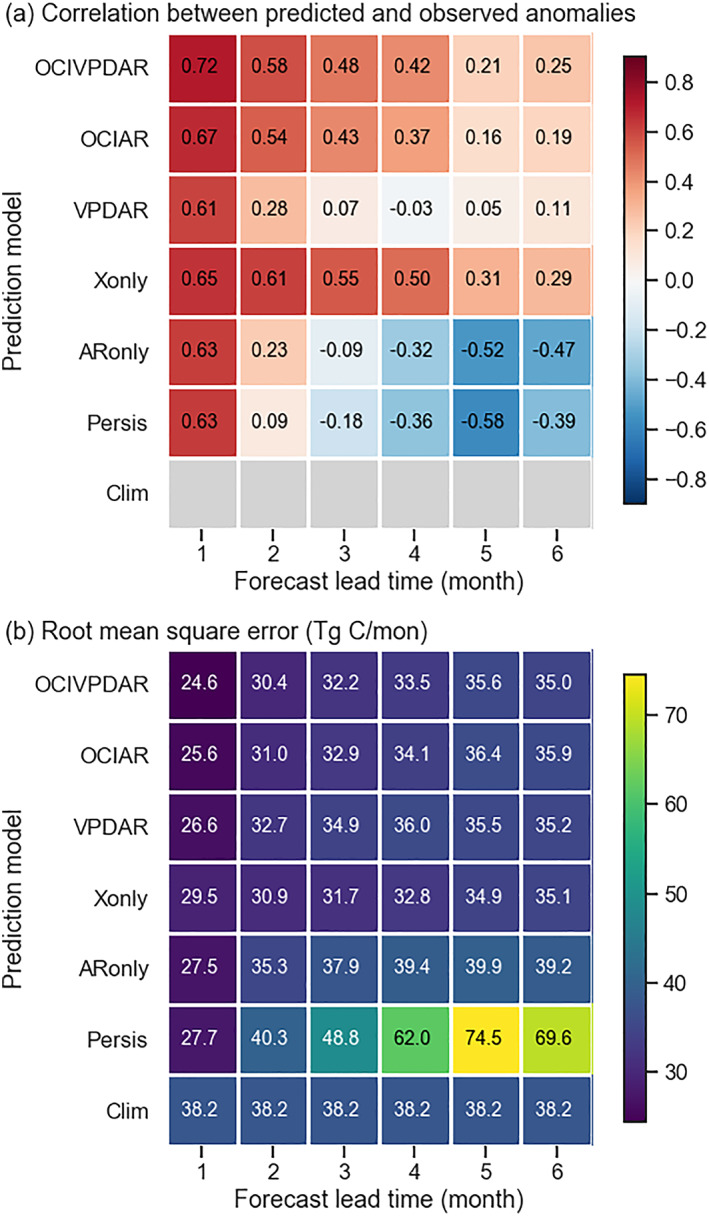
Global model performance as a function of model structure and forecast lead time (1–6 months) during the model prediction interval from 2015 to 2018. (a) The correlations between observed and modeled fire emission anomalies. (b) The root‐mean‐square errors of fire emissions predictions.

**Figure 11:**
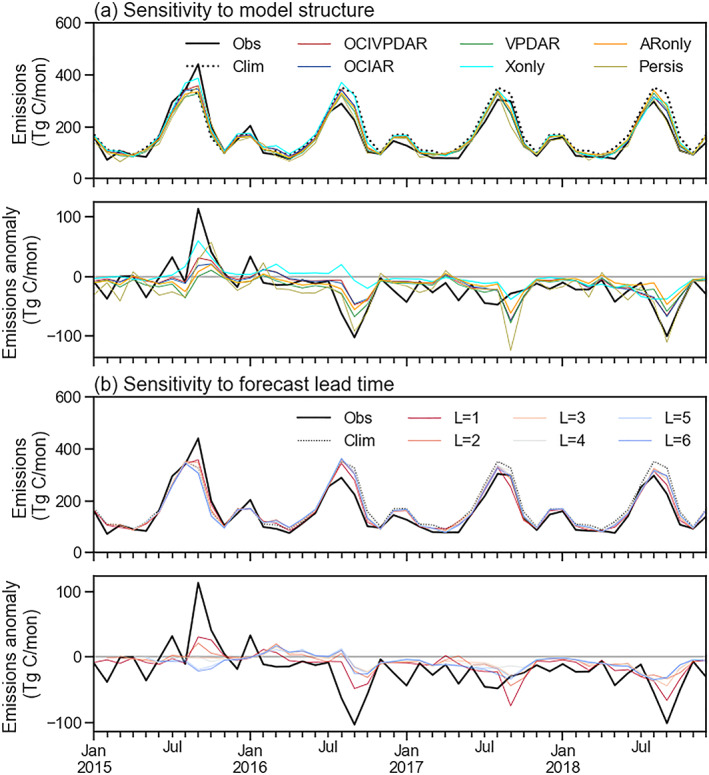
Time series of global sum fire emissions and forecast model estimates for the evaluation period (out‐of‐sample interval, 2015–2018). (a) Sensitivity of modeled fire emissions and anomalies (relative to the 1997–2014 climatological mean) to model type (for forecast lead time of 1 month). (b) Sensitivity of modeled fire emissions and anomalies to forecast lead time (*L*) using the *OCIVPDAR* model. Solid and dashed black lines show data series of GFED4s and the climatological mean, respectively.

Performance indicators, including the correlation coefficient of the predicted anomaly with the observations and the RMSE of predicted fire emissions, showed considerable variability in forecast abilities of models with different predictors and forecast lead times (Figure 10). In general, the ARIMAX‐based models (OCIVPDAR, OCIAR, VPDAR, Xonly, and ARonly) produced better predictions than the simpler models based on anomaly persistence or a climatology of past fire emissions. For a 1 month forecast lead, our reference model (*OCIVPDAR*) resulted in a small underestimation (6%) of global fire emissions, and explained about 52% of the temporal variability in the global sum fire emissions during the model evaluation period (Figure 10). The model also captured broad patterns of climate‐driven fire anomalies, with higher emissions during the 2015 El Niño year, and lower emissions in subsequent years (Figure 11a).

The *OCIVPDAR* model, which includes both OCI and VPD as exogenous predictors, showed the best overall performance among the ARIMAX models, with lowest RMSE and highest correlation with the observations (Figure 10). For short lead times, the two models created by excluding autoregression (*Xonly*) or exogenous predictors (*ARonly*) had similar scores, suggesting similar contributions of information from climate and the past time series of fire emissions.

As the forecast lead time increased, model performance generally declined (Figure 10). Forecast skill from the *OCIVPDAR* model tended to become closer to that of the *Clim* model at longer forecast lead times (Figures 11b). For the *ARonly* model, the performance degraded faster, and the RMSE from this model was comparable to that from the *Clim* model for a forecast lead time of about 4 months. Models with OCIs as a predictor had a lower rate of degradation, likely reflecting the importance of low frequency, wave‐like dynamics of climate information associated with OCI teleconnections (Diaz et al., 2001).

The model with an OCI as the exogenous predictor (*OCIAR*) had slightly better performance than the model with VPD as the only exogenous predictor (*VPDAR*) at shorter lead times, and this difference increased at longer lead times. Overall, the inclusion of an OCI component (e.g., the *OCIVPDAR* and *OCIAR* models), in addition to AR and VPD, improved model performance at longer lead times.

#### 5.2.2 Spatial Distribution of Model Performance

**Figure 12:**
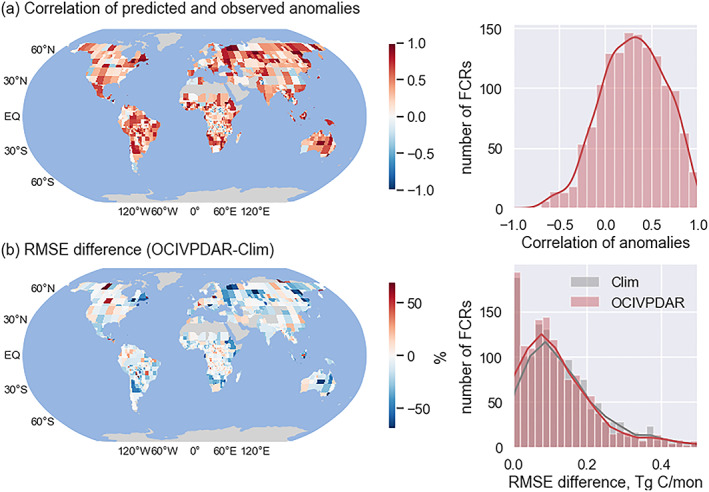
Comparison of the performance of the *OCIVPDAR* model (at forecast lead time of 1 month) to that of the *Clim* model during the evaluation period (2015–2018). (a) Correlation coefficient between the modeled and GFED4s emissions anomalies (with the climatological values subtracted). (b) The root‐mean‐square error (RMSE) difference between the *Clim* model and the *OCIVPDAR* model, reported as percent change. Positive correlations and negative RMSE differences indicate that the *OCIVPDAR* model outperformed the *Clim* model. Left panels show global maps of indicators, while the right panels show the histograms over all fire cohesive regions (FCRs). See Table 3 for detailed description of different forecast models.]

Model performance was not uniform across different continental‐scale regions (Figure 12). The OCIVPDAR model was able to successfully predict fire emissions anomalies in many important high‐fire regions, as indicated by mostly positive correlation coefficients between predicted and observed anomalies, and a lower RMSE between predicted and observed fire emissions than an RMSE constructed from the observations and a climatology of past fire observations. Regions with high model performance included eastern Canada, eastern boreal Asia, equatorial Asia, eastern Australia, eastern and southern Africa, and southern South America.

By comparing model performance in the model evaluation period, we were able to assess the relative importance of autoregression, large‐scale climate pattern, and local meteorology to forecast performance in different regions (Figure S11). Using RMSE as the standard, OCI had a larger contribution in regions where global and regional modes of climate variability are known to have large impacts, such as pan‐tropical regions where fires response to ENSO in a predictable sequential way (Chen et al., 2017), and other hot spots where burned area has a relatively high degree of predictability due to their coherent response to OCIs (Chen et al., 2016). Similarly, autoregression was more important over most regions for a 1 month forecast lead, whereas climate became increasingly important for a 3 month lead time. Maps of the best models for each FCR at forecast lead time of 1 month and 3 months are shown in Figure S11c.

#### 5.2.3 Model Evaluation in Regions of High Biomass Burning

**Figure 13:**
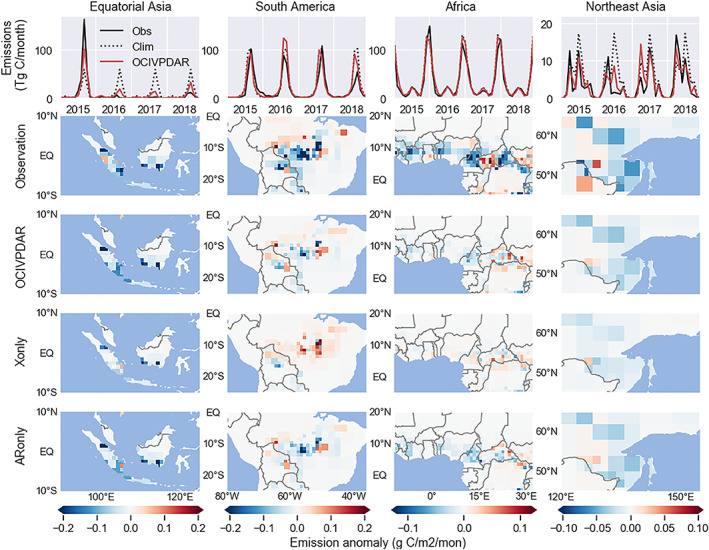
Comparisons between GFED4s and modeled fire emissions in four high‐fire regions during the evaluation period (2015–2018). Top panels show monthly time series of fire emissions from observations (black), the *Clim* model (dotted), and the *OCIVPDAR* model (red). Other panels show the spatial maps of fire emissions anomalies (with the climatological means subtracted) averaged over 2015–2018. The predictions are from models at forecast lead times of 1 month.

Here, we specifically examined how the components of the ARIMAX model, including different endogenous and exogenous predictors, contributed to model performance in several regions with high fire activity (Figure 13).

In equatorial Asia, the models with a 1 month lead time successfully captured the extremely high fire season following the strong El Niño in 2015, and the lower than normal patterns in the following 3 yr. The spatial patterns of the emission anomalies were also reproduced by the modeling system with 1 month forecast lead. Both models using climate only (*Xonly*) and autoregression (*ARonly*) were able to capture the general pattern of emissions anomalies in this region. For this short lead time, the autoregression component contributed more to the negative fire emissions anomaly in southern Sumatra.

The cross validation indicates the full *OCIVPDAR* model captured some of the spatial pattern but overestimated emissions in tropical South America during the 2016 and 2018 fire seasons. Specifically, the model using climate variables as the only predictors (and excluding the ARIMA component) (*Xonly*) was not able to capture the magnitude of reduction of fires in the arc of deforestation in South America. A decreasing trend in human use of fires in this region has been reported (Aragao & Shimabukuro, 2010; Chen, Velicogna, et al., 2013), which may not be captured by the climate‐only model, resulting in poor predictions. In tropical South America, the AR component of the model was critical for improving the performance of the full model.

In Africa, the year‐to‐year changes in fire emissions are small, and statistical approaches may not have the capability to capture variability driven by a complex set of human and environmental drivers. We observed that climate‐only model led to some positive anomalies near the Congo basin, while the autoregression contributed to the negative anomaly prediction in western Africa. Although the spatial pattern of the predicted long‐term anomalies generally agreed with observations, the magnitudes of the anomalies were considerably underestimated.

Using region‐specific ARIMAX model to forecast fire emissions in extratropical regions is expected to be more difficult because fires in these regions are often more sporadically distributed in space, and less influenced by tropical OCIs. Nevertheless, the OCIVPDAR model in our forecast system was able to reproduce the main spatial pattern of fire emissions anomalies in many high latitude regions, including eastern Siberia region in northeast Asia shown in Figure 13. We also found both climate and autoregression contributed to the simulated negative anomaly across this region.

## 6 Discussion

### 6.1 S2S Prediction for Fires That Respond to Multiple Climate and Human Drivers

Climate and humans are two main regulators for the distribution and variability of global fires (Knorr et al., 2016). However, our ability to assess and separate climate and human influences on the historical fire record is still limited, and an even more challenging problem is to account for both effects in predicting future fire occurrence. In this study, we attempted to implicitly account for both sets of drivers, including long‐term secular trends, by using the ARIMAX model framework.

OCIs have been widely used in previous efforts to build seasonal fire forecasts, and they can be better predictors of ecological processes than local climate (Hallett et al., 2004). However, local meteorological variables may provide additional information for fire forecasts in some regions, where large‐scale influences are not well represented by a single OCI. In this study we included both OCI and local VPD as exogenous predictors. We found comparable forecast skill for models that only considered large‐scale climate or local meteorology at short lead times, and an increasingly important role for OCIs at longer lead times (Figure 10). We also optimized the time delay between the exogenous variables and predicted emissions in each region. As a result, our model was able to capture climate impacts through different mechanisms (including shorter‐term impacts of climate on fuel moisture and longer‐term impacts on fuel availability), as well as spatial variations in the importance of these different influences (Figure 7).

Humans modify fire ignition as well as fuels, including loads, moisture, continuity. Anthropogenic greenhouse gas emissions are also driving new extremes in fire weather, contributing to an intensification of fire regimes in many temperate and boreal biomes (Abatzoglou & Williams, 2016; Flannigan et al., 2013). Although humans play a central role in regulating the fire regime of important high‐fire regions, it has been challenging to represent these interactions in global fire models (Lavorel et al., 2007). It is even more challenging to integrate human predictors into a statistical model for S2S fire forecasting. Reliable indictors exist for describing the influence of humans on the spatial structure of fire activity, including population, road density, livestock, and cropland area (Andela et al., 2017; Faivre et al., 2014), yet none of these variables are well suited for understanding how fire activity may vary within a single contiguous region over a 1–6 month time scale typical of S2S forecasting systems. For this reason, we opted not to include explicit human predictors in our modeling system. Instead, we note that our modeling framework can implicitly account for some human impacts on fire regimes. For example, by having spatially explicit optimization for the ARIMAX model in over 1,300 individual regions and separating these regions along country boundaries, we partially accounted for spatial differences in land management practices. In each FCR, the differencing component (I in the ARIMAX) allowed for the inclusion of fire responses to slowly varying human (and potentially climate) influences in our forecasting system. In the forecasts, these trends were implicitly assumed to continue. We found that, in regions with significant long‐term trends during the study period (such as North Africa and the deforestation arc in South America), the inclusion of differencing appeared to improve our forecasts, as expected. Some fast‐changing human impacts, such as a change in policy regarding fire use, were also implicitly included in our system by allowing the monthly adjustment in parameters (*α*_*i*_) of the autoregression (AR) component. However, we note that human drivers to fires in the real world are more complex and extremely difficult to predict. An incomplete representation of human‐fire interactions certainly contributed to the unexplained variability in our system.

### 6.2 S2S Prediction That Combines Long‐Term Ecological Memory and Near‐Real‐Time Climate Data

Our work provided evidence that recent fire observations contain considerable information about the likelihood of future fire during the same fire season. This is likely a consequence of ecological memory: many climate and human factors that create conditions more or less favorable for fire are set before the onset of the fire season and change only very slowly on S2S time scales. Yet, near‐real‐time fire data are rarely included in forecasting efforts. The AR and MA components in our model system explicitly used recent fire measurements and errors to improve forecasts beyond the capability of climate‐only models in many regions.

One of the important contributions of this study is that we created a global system that can determine the region‐specific relative contribution of the above mentioned drivers. Using historical data, we individually tailored the optimal OCI region and lag time, the optimal lag for VPD, the *d* value representing the differencing order, and the *p* and *q* values representing key controls on the auto regression and moving average components of the model for each FCR. Thus, the ARIMAX modeling framework allowed us to select the best form of the statistical model for each region in a consistent manner.

### 6.3 Improving S2S Fire Prediction Using Dynamical Seasonal Forecasting Models

To account for climate impacts, our ARIMAX‐based forecast system allowed for lagged predictors from OCIs and VPD. Only the OCI and VPD values observed at or before the date of the forecast (and hence before the target prediction date) were used in the design of our model. It is important to note in this context that past work has explored the use of climate model projections instead of observations to predict fires (e.g., Spessa et al., 2015). The skill of such predictions, to a large extent, depends on the accuracy of the climate model seasonal forecast, which also decays with increasing forecasting lead time. For example, prediction of El Niño typically shows limited skill before the preceding boreal spring (the so‐called “spring barrier”) (Webster & Hoyos, 2010), although recent advancements have provided some potential approaches to overcome this barrier (Izumo et al., 2010; Masuda et al., 2015).

An important next step in this context is to examine if (and where) dynamical seasonal forecasts of OCIs and fire weather variables from state‐of‐the‐art climate models can improve the predictability of our system. A key challenge in this regard is understanding how the information content of different surface climate variables that influence fine fuel amount and fuel moisture degrades as a function of forecast lead time in different regions. If, for example, ENSO and other indices are predicted with more skill at longer lead times than VPD or precipitation in high‐fire regions, these ocean variables may be better suited for initial integration within an S2S fire model.

### 6.4 Complexity of the Fire Forecast

While our model was able to capture major patterns of fire variation, the skill of the forecast was likely limited by both the simple structure of the model, uncertainties in the driver data sets, and insufficiency in the quality and temporal duration of the fire emissions data we were attempting to predict. Below we summarize several physical processes and statistical properties that may contribute to the uncertainties of the fire forecasts from our modeling system. Addressing many of these uncertainties is an important direction for future work.

#### 6.4.1 Ocean Teleconnections

In the current system, for each region we selected one of the 14 OCIs to account for the effect of large‐scale climate patterns on fire variability. While using an operationally produced single OCI can simplify the forecast and reduce the risk of over fitting, the information from the climate system for the fire forecast may be not optimized. For instance, combining two OCIs from different oceans has been shown to improve model forecast of burned area in many regions (Chen et al., 2016). An alternative approach would be to use machine learning to find an optimal ocean region for each specified FCR, and to subsequently use the SST time series from this region to construct the forecasting model. This approach for tailoring the SST information for each fire prediction region would provide for more flexibility in picking the proxy for the influence of large‐scale climate and improve forecast skill.

The OCIs used in this study are produced operationally from the NOAA Optimum Interpolation (OI) SST data. The SST time series are constructed from a combination of satellite observations and in situ measurements (Reynolds et al., 2002). It is our assessment that errors introduced from biases or incomplete sampling of SSTs in OCI regions from the NOAA processing are relatively small compared to other sources of error in our modeling system (see discussion of VPD and GFED4s emissions estimates below). A key challenge, however, from the perspective of building an operational S2S fire prediction system is the latency of OCI data generation. Currently the OCIs were produced weekly or monthly with a latency of 2–4 weeks.

#### 6.4.2 Local Fire Weather

VPD is a good indicator for atmospheric and fuel moisture, and has been shown to be correlated to fire occurrence in many ecosystems. However, its influence on fire is mostly limited to shorter time horizons. The rapid drop in the performance of our VPD‐only model (shown in Figure 10) confirmed the inadequacy of using VPD for forecasts with longer lead times. Fires are probably also less sensitive to changes in VPD in regions where fuel amount is a primary limiting factor, rather than fuel moisture. Other local weather‐related parameters, including factors that contribute to changes in vegetation productivity or soil moisture in the preceding growing season, may provide more useful information for the purpose of fire forecast in some regions. An example is that wildfire activity in western United States has been found to be strongly associated with local surface air temperatures and the timing of spring snowmelt (Westerling et al., 2006).

We also note that there are inherent limits to the quality of reanalysis estimates for surface temperature and relative humidity needed to estimate VPD. Here we use estimates from MERRA‐2 in order to provide complete global coverage. Uncertainties in this reanalysis product have been evaluated by Yi et al. (2011) and Bosilovich et al. (2017). Biases and errors of both surface temperature and VPD vary with latitudes and are generally larger in the southern hemisphere. From an operational S2S prediction perspective, near‐real‐time analysis products are likely to have even higher levels of uncertainty, and may limit the use of this data stream for prediction, particularly in areas where radiosondes and surface climate networks are relatively sparse.

#### 6.4.3 High‐Frequency Variability and Noise

Both fire and climate measurements have uncertainties, leading to the presence of noise in the data time series. It is often difficult to separate this noise from real high‐frequency variability, such as month‐to‐month changes in precipitation. For fire emission time series that often have a strong seasonal cycle, the noise in low‐fire months can be large as a consequence of a small number of satellite fire detections, although the use of high orders of *p* and *q* in the ARIMAX model may have allowed us to smooth out some of this noise. The performance of our modeling system may suffer from overly tracking these “false” variations in climate predictors (especially for local VPD), since we only used OCI and VPD time series sampled using a predetermined optimal lag time.

#### 6.4.4 Year‐to‐Year Variations in Fire Seasonality

Considering that fire emissions in most regions have distinctive seasonal cycles, we first transformed the data and removed the seasonal component using an additive decomposition. An intrinsic assumption to our forecasting approach was that the seasonal component remained the same over time. Although this assumption is likely valid in many regions, our model is not able to forecast a sudden change in fire regime due to, for example, a change in a country's fire regulation policy or climate‐induced changes in the onset and duration of the fire season. The length of the fire season and the monthly distribution of fire emissions are also linked with the severity of the fire season. The seasonal cycle derived from historical observations can also be less reliable in regions with infrequent fire occurrence (e.g., Level 8r FCRs). Developing an approach that allows for the temporal evolution (and interannual variability) of the shape of the fire season may improve forecasting skill, although this will also require access to longer and higher quality time series of fire observations.

#### 6.4.5 Regional Heterogeneity Within a Forecast Region

In our analysis, the monthly sum of GFED4s emissions in each FCR was considered as the target for prediction. But the fire response to predictors may have spatial gradients within a region, likely arising from small‐scale variations in surface properties and local climate, as well as different types of ecosystems. For example, forest fires, savanna fires, and deforestation fires can be simultaneously present in a single FCR in the southern Amazon. Their seasonality, trend, and sensitivity to climate in wet and dry seasons are quite different (Andela et al., 2019; Chen, Morton, et al., 2013; Morton et al., 2013) and may require models that are individually tuned for each land cover type. Developing such a model might be useful in regions where different fire types changing, for example, in response to a shift in the land use frontier. Ecoregions represent geographical aggregations of natural fauna and flora, and relatively uniform fire regimes regulated by climate and vegetation (Pausas & Ribeiro, 2013). A hybrid approach by combining ecoregion information with our fire data‐driven FCR approach may improve the aggregation of homogenous regions for fire prediction, particularly in temperate and boreal forest regions that burn infrequently. Using higher resolution fire detections from new satellite instruments (Schroeder et al., 2014) may further help to resolve some of the within‐region variability in fire dynamics.

#### 6.4.6 Synergic Variability in Nearby Regions

We performed model fitting and forecasting separately for individual FCR in the current system. This region‐specific approach can mitigate the spatial heterogeneity problem mentioned above (section 6.4.5), but at the same time may be sensitive to sources of nonphysical noise. Since fire activity is typically associated with synoptic‐scale weather events, we expect that time series of fire emissions in nearby regions may share similar temporal patterns of variability. More advanced methods, such as multivariate ARIMAX models that consider two or more regions simultaneously, may systematically examine synergistic variability in nearby regions, and fuse near‐real‐time information from neighbors into the forecasting model.

#### 6.4.7 Global Fire Emissions Data

There are multiple fire measurements that can be used to characterize fire variability, including active fire detections, burned area, fire radiative power, fire emissions, and concentration of fire emitted trace gases and aerosols. In this study, we trained and evaluated our statistical models with the GFED4s fire emissions data. While GFED used multiple sources of satellite fire detections during different periods, it was designed to provide a spatially and temporally consistent set of emissions (van der Werf et al., 2017) and has been partly validated through comparison to other independent estimations (Pan et al., 2020) as well as measurements of fire emitted trace gases (e.g., Pechony et al., 2013) and aerosols observations (e.g., Wiggins et al., 2018). In the past two decades, the quality of GFED has been improved by leveraging new satellite data products, algorithm advances, and field measurements (van der Werf et al., 2017). A new version of GFED, which has been in development in a separate project, will further reduce uncertainties and extend global fire emissions time series through the next decade.

## 7 Summary and Conclusions

The inherent stochastic nature of fire occurrence over a wide range of spatial and temporal scales initially calls for a statistical rather than a deterministic approach for S2S prediction. Before the satellite era, people often relied on historical administrative records to get early warning information at a regional scale (e.g., Stocks et al., 1989). Global time series of satellite‐derived burned area and fire emissions data now span more than two decades (van der Werf et al., 2017), making it possible to derive predictions of global fires for various lead times by means of statistical methods, and to test the performance of different models. By using a time series of monthly fire emissions and tailoring individual models within each of 1,380 FCRs, we established a global fire emissions forecasting system. This system, building on a statistical ARIMAX modeling framework, was able to handle different sources for predicting fire emissions over forecast lead times of 1 to 6 months. Our results indicate that the joint use of past fire observations and climate data can improve our understanding of fire processes and provide reliable S2S forecasts of monthly fire emissions in many regions. The modeling system we describe here may provide the foundation for an experimental, operational global fire forecasting system, creating a bridge between near‐real‐time predictions of fire weather and seasonal fire outlooks. Information from such a system may be useful in helping fire managers in many areas adapt to changing fire threats as a consequence of climate and other forms of global environmental change.

## Supporting information

Supporting Information S1

## Data Availability

The data sets used in this paper are all in the public domain and citations are provided within the text. These include (1) Global Fire Emissions Database Version 4s (GFED4s), available at the GFED website (http://www.globalfiredata.org) and archived at the NASA ORNL DAAC; (2) Ocean Climate Indices (OCIs), available at the NOAA state of the Ocean website (http://stateoftheocean.osmc.noaa.gov/sur/); and (3) The MERRA‐2 climate data, available at NASA GES DISC (https://disc.gsfc.nasa.gov/).
