# Supplementary material for: Forecasting Global Fire Emissions on Subseasonal to Seasonal (S2S) Time Scales
Source: J Adv Model Earth Syst. 2020 Sep 18;12(9):e2019MS001955. doi: 10.1029/2019MS001955 (PMC7540459; doi:10.1029/2019MS001955)
Supplement: Supplementary file 1 — Supporting Information S1 [file JAME-12-e2019MS001955-s001.pdf]

AGU PUBLICATIONS

Journal of Advances in Modeling Earth Systems

Supporting Information for

Forecasting global fire emissions on sub-seasonal to seasonal (S2S) timescales

Yang Chen[1], James T. Randerson[1,2], Shane R. Coffield[1], Efi Foufoula-Georgiou[1,2], Padhraic Smyth[3], Casey A. Graff[3], Douglas C. Morton[4], Niels Andela[4], Guido R. van der Werf[5], Louis Giglio[6], and Lesley E. Ott[7]

[1] Department of Earth System Science, University of California, Irvine, CA, 92697, USA
[2] Department of Civil and Environmental Engineering, University of California, Irvine, CA, 92697, USA
[3] Department of Computer Science, University of California, Irvine, CA, 92697, USA
[4] Biospheric Sciences Laboratory, NASA Goddard Space Flight Center, Greenbelt, MD, 20771, USA
[5] Faculty of Sciences, Vrije Universiteit Amsterdam, 1081 HV Amsterdam, The Netherlands
[6] Department of Geographical Sciences, University of Maryland, College Park, MD, 20742, USA
[7] Global Modeling and Assimilation Office, NASA Goddard Space Flight Center, Greenbelt, MD 20771, USA

Contents of this file

Table S1
Figures S1 to S11

Table S1. A list of acronyms and abbreviations used in this paper.

Name	Explanation
ARIMAX	Autoregressive Integrated Moving Average model with eXogenous variables
ACF	AutoCorrelation Function
FCR	Fire Cohesive Region
GFED	Global Fire Emissions Database
MERRA	Modern-Era Retrospective analysis for Research and Applications
OCI	Ocean Climate Index
NINO4	Niño 4 SST Index (160°W-150°W, 5°S-5°N)
NINO34	Niño 3.4 SST Index (170°W-120°W, 5°S-5°N)
NINO3	Niño 3 SST Index (150°W-90°W, 5°S-5°N)
NINO12	Niño 1+2 SST Index (90°W-80°W, 10°S-0°)
PDO	Pacific Decadal Oscillation Index (North of 20°N)
TNI	Trans-Niño Index (NINO12 - NINO4)
TNA	Tropical Northern Atlantic SST Index (55°W-15°W, 5°N-25°N)
TSA	Tropical Southern Atlantic SST Index (30°W-10°E, 20°S-0°)
NAT	North Atlantic Tropical SST Index (40°W-20°W, 5°N-20°N)
SAT	South Atlantic Tropical SST Index (15°W-5°E, 20°S-5°S.)
TASI	Tropical Atlantic SST Index (NAT – SAT)
AMO	Atlantic Multidecadal Oscillation Index (0°-70°N)
SWIO	South Western Indian Ocean SST Index (31°E-45°E, 32°S-25°S)
WTIO	Western Tropical Indian Ocean SST Index (50°E-70°E, 10°S-10°N)
SETIO	Southeastern Tropical Indian Ocean SST Index (90°E-110°E, 10°S-0°)
DMI	Dipole Mode Index
PACF	Partial AutoCorrelation Function
RMSE	Root Mean Square Error
S2S	Sub-seasonal to Seasonal
SST	Sea surface temperature
VPD	Vapor Pressure Deficit

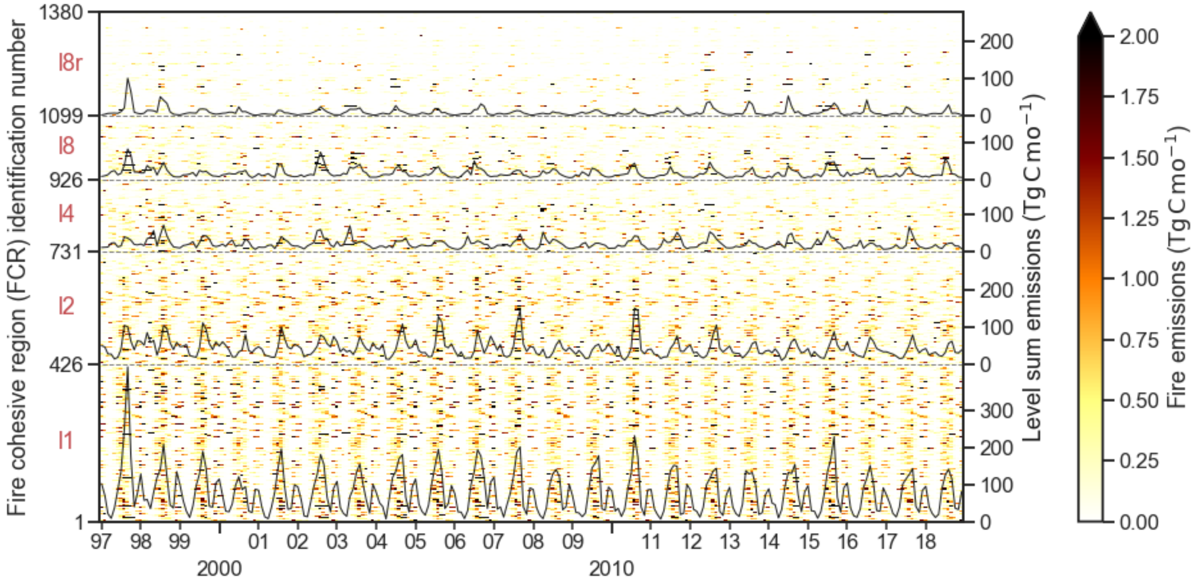

Figure S1. Monthly fire emissions (shown in color shades, stacked vertically) in each fire cohesive region (FCR) defined in this study. The sums of emissions for each FCR resolution level are shown as black lines. FCRs at level 1 (l1) had a 1° x 1° spatial resolution, FCRs at l2 had a 2° x 2° resolution, l4 had a 4° x 4° resolution, and l8 and l8r had an 8° x 8° resolution.

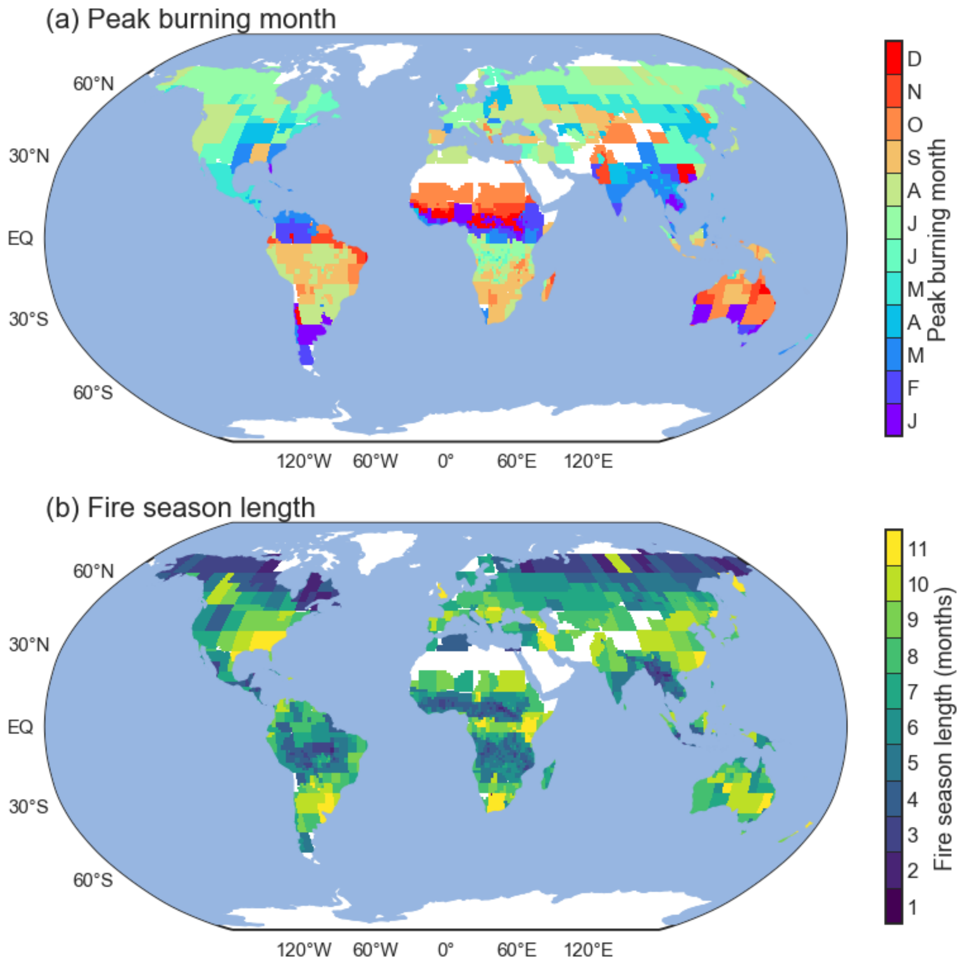

Figure S2. Global maps of (a) peak burning month and (b) fire season length for each FCR used for fire forecasting. The fire season is defined as a minimum number of continuous months in which the fire emissions comprised at least 95% of the annual total during 1997-2014.

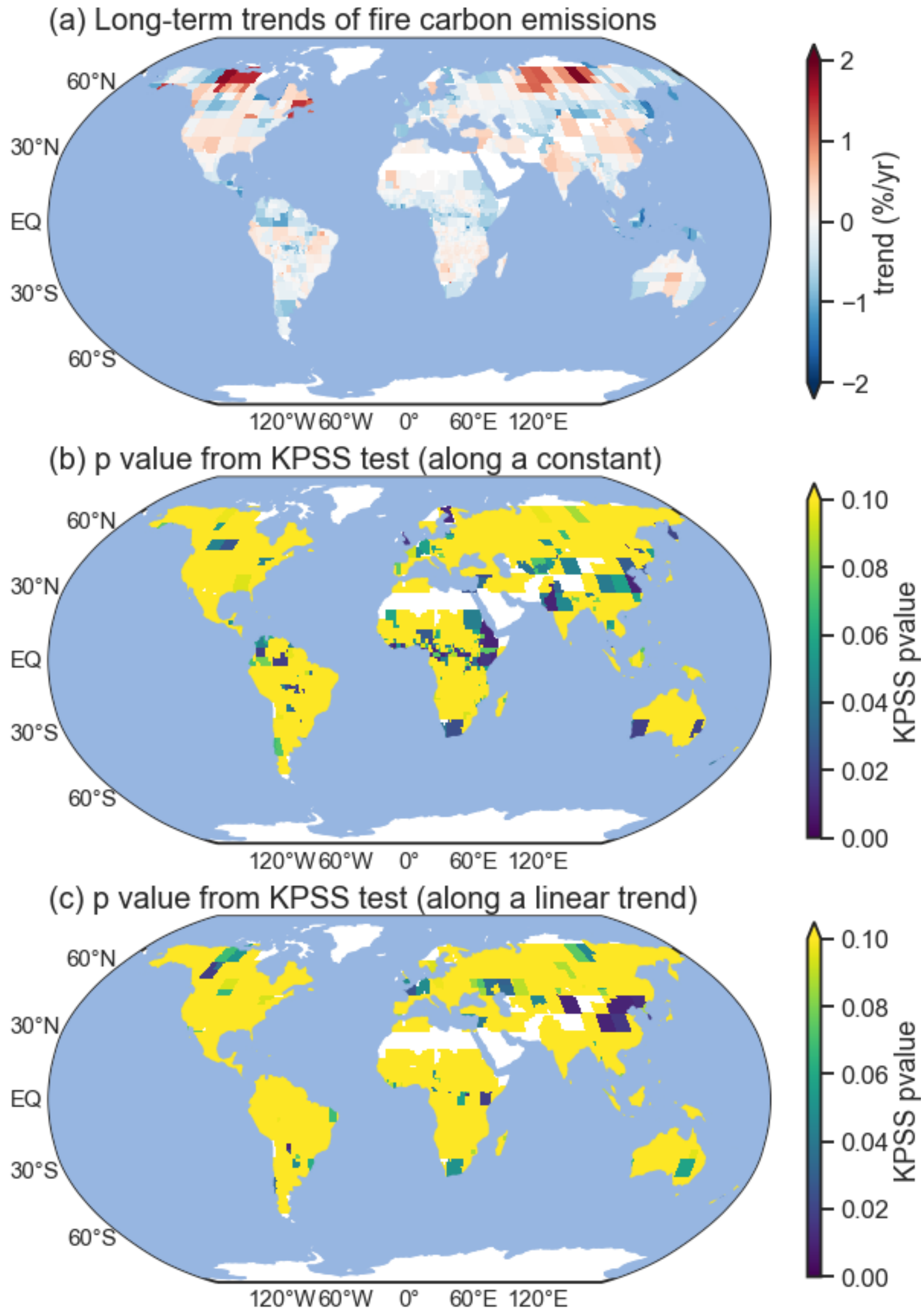

Figure S3. Global maps of results from fire emission stationarity tests. (a) Fire emissions long-term trend in percent per year, (b) and (c) show *p* values from KPSS tests, with the null hypothesis that the time series is stationary along a constant or along a deterministic trend, respectively.

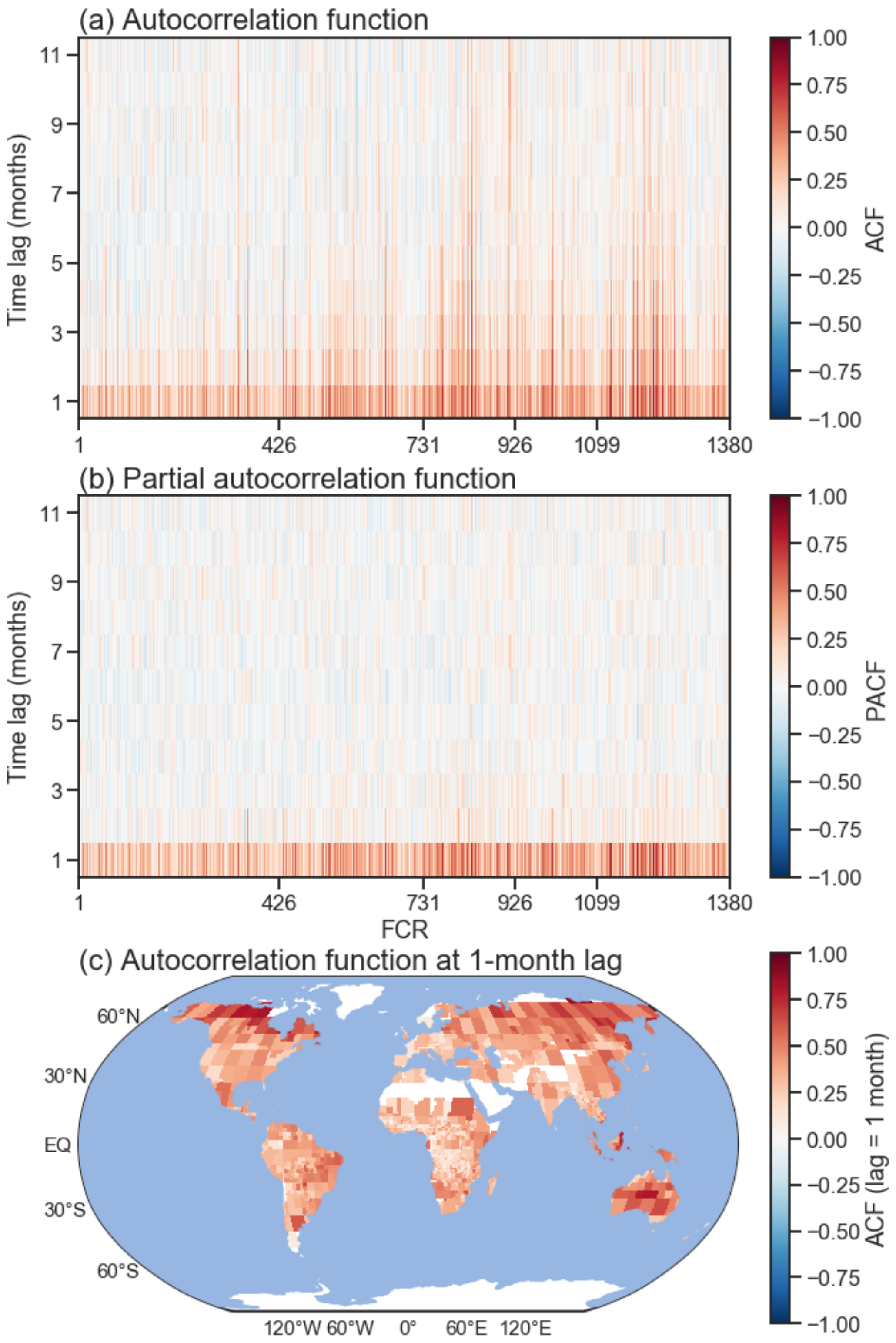

Figure S4. Autocorrelation function (ACF) and partial autocorrelation function (PACF) of fire emission anomalies at different FCRs. (a) Autocorrelation coefficients as a function of lag time, (b) Partial autocorrelation coefficients as a function of lag time, (c) Global map of autocorrelation coefficients for a lag time of 1 month.

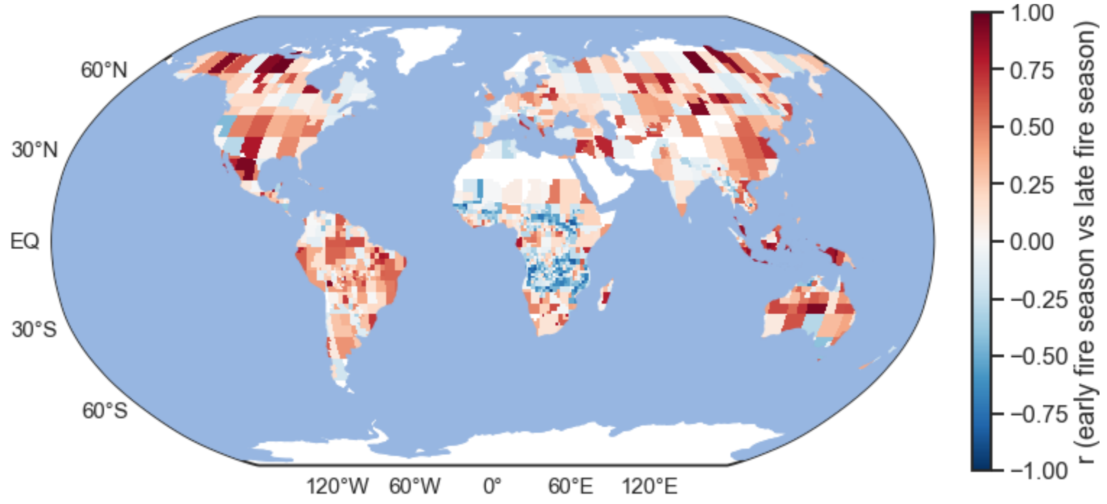

Figure S5. Global map of correlation between emissions in early and late stages of the fire season. The correlation was calculated using 18 (1997-2014) annual pairs of points in each region, with each pair representing fire emission anomalies summed over the early fire season (1-6 months before the peak burning month) and over the late fire season (1-5 months after the peak burning month) in a given year.

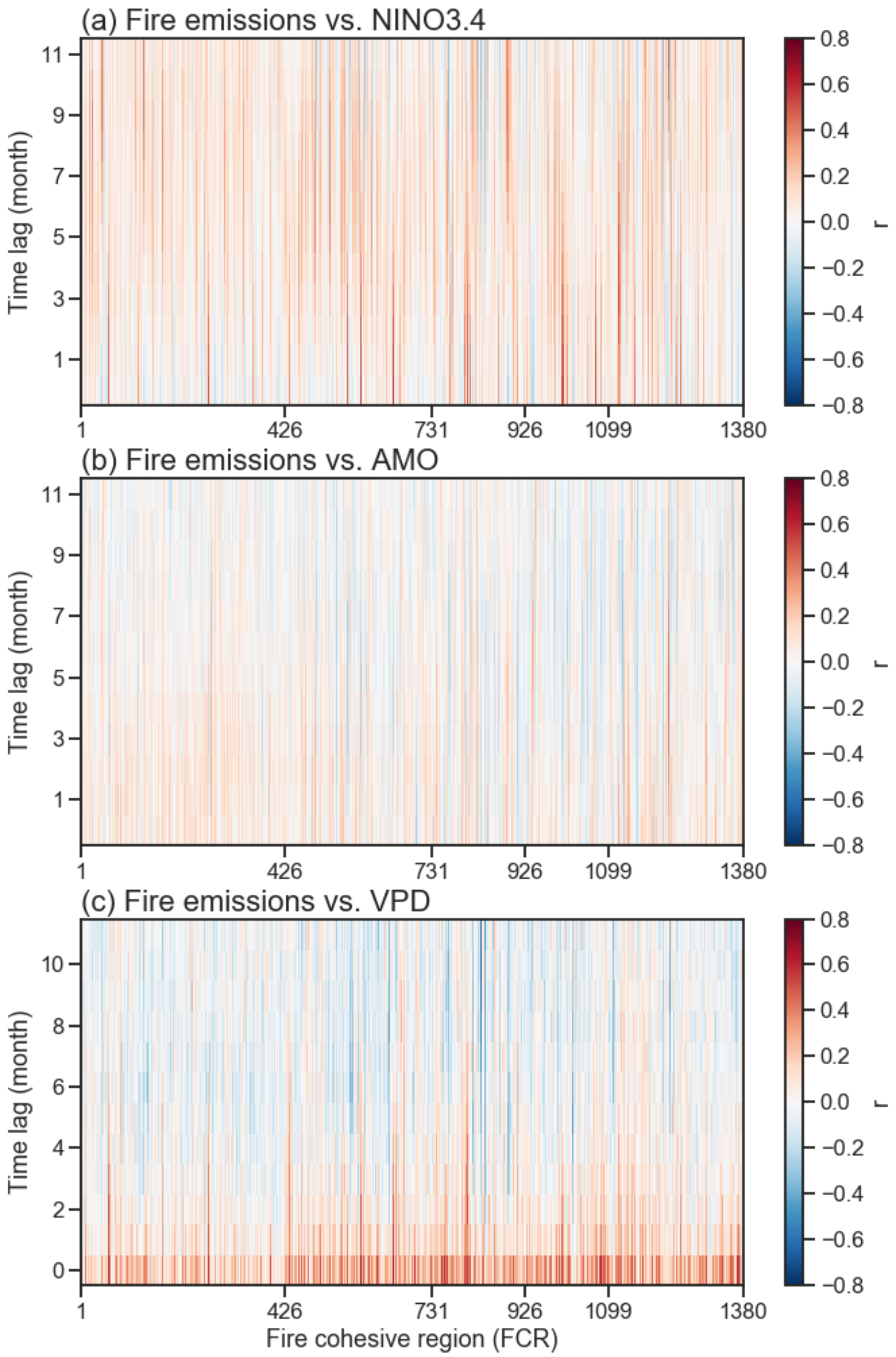

Figure S6. Correlation of fire anomalies with two OCIs (NINO3.4 and AMO) and VPD for different lag times.

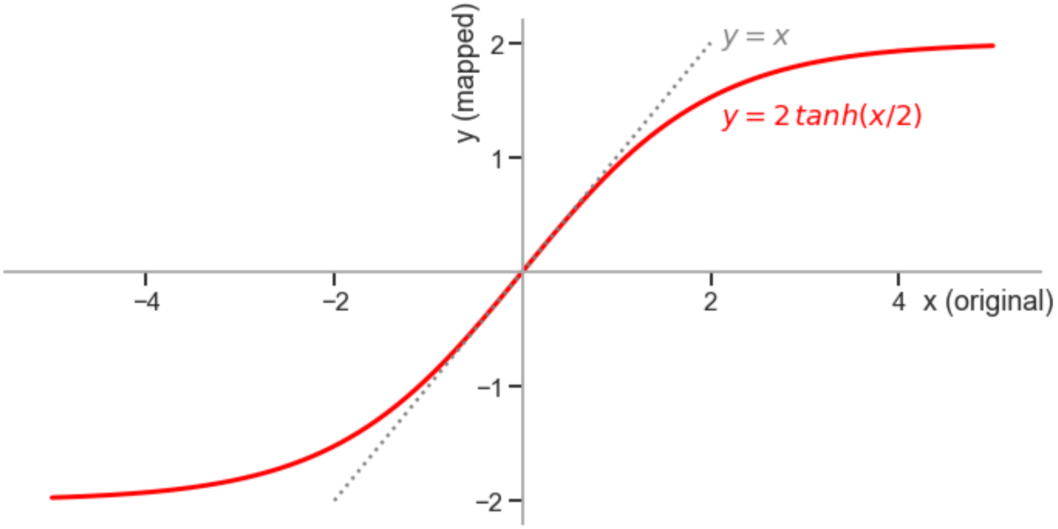

Figure S7. Mapping the data using a hyperbolic tangent function that conserves the relative relationship and also limits the data within a range of -2 to 2.

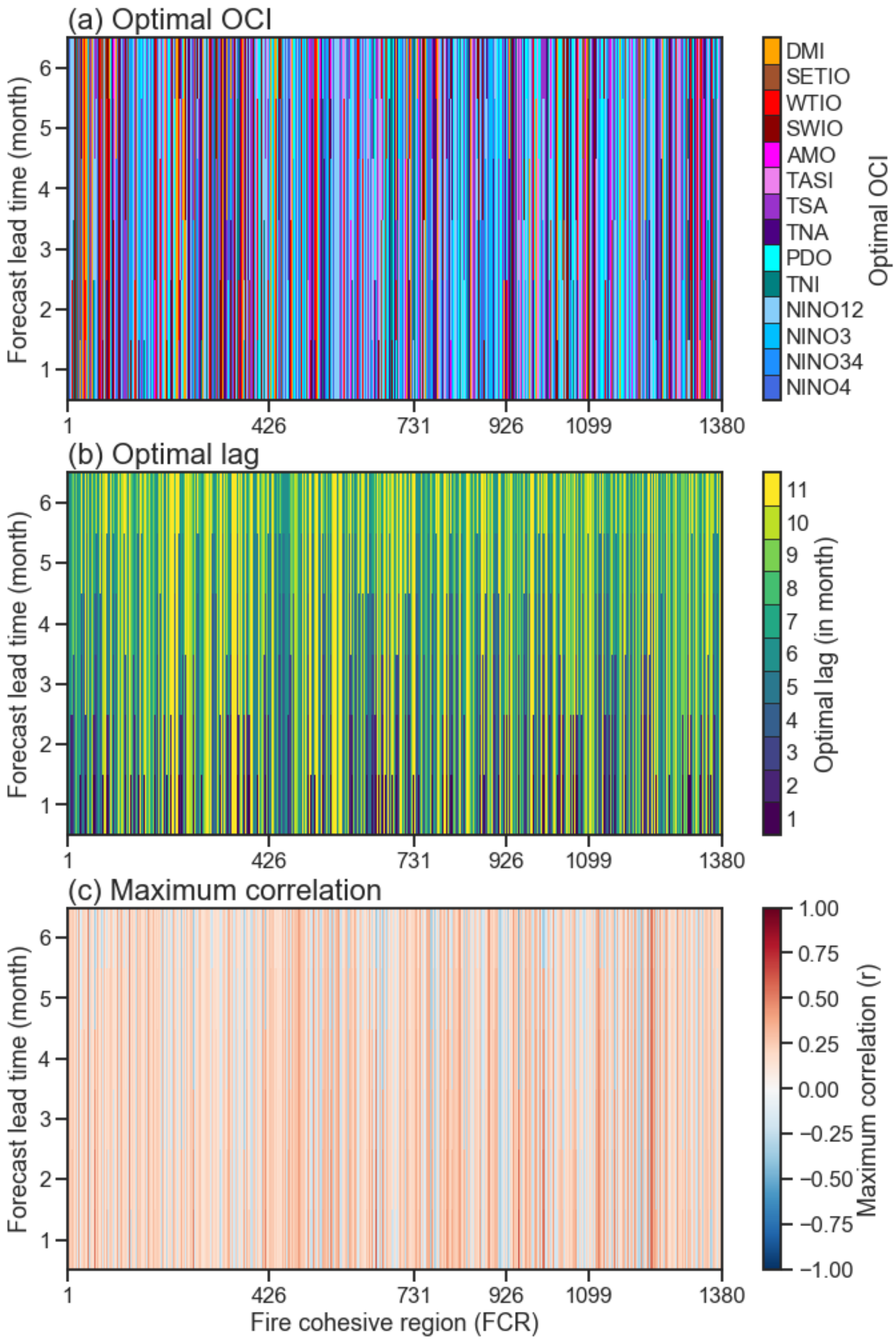

Figure S8. Optimal OCI as a function of forecast lead time (1-6 months). (a) The optimal OCI for emission prediction in each FCR, (b) The optimal lag time (in months) for the optimal OCI as shown in (a), (c) The correlation between the emission anomalies and the optimal OCI time series (with optimal lag).

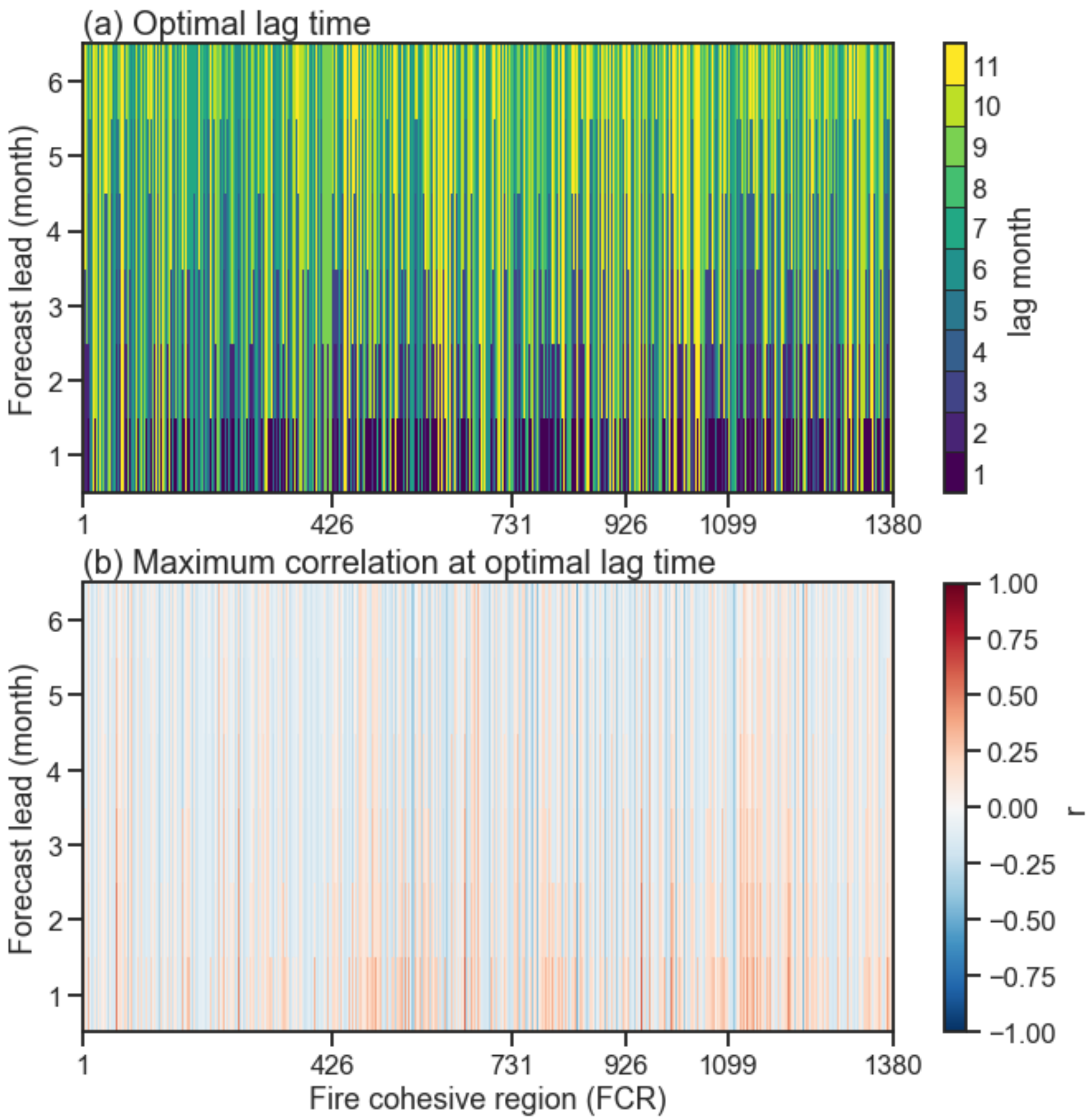

Figure S9. Optimal VPD lag time as a function of forecast lead and Fire cohesive region (FCR) identification number. (a) The optimal lag time (in months) for VPD, (c) The correlation between the emissions anomalies and the VPD time series (with optimal lag).

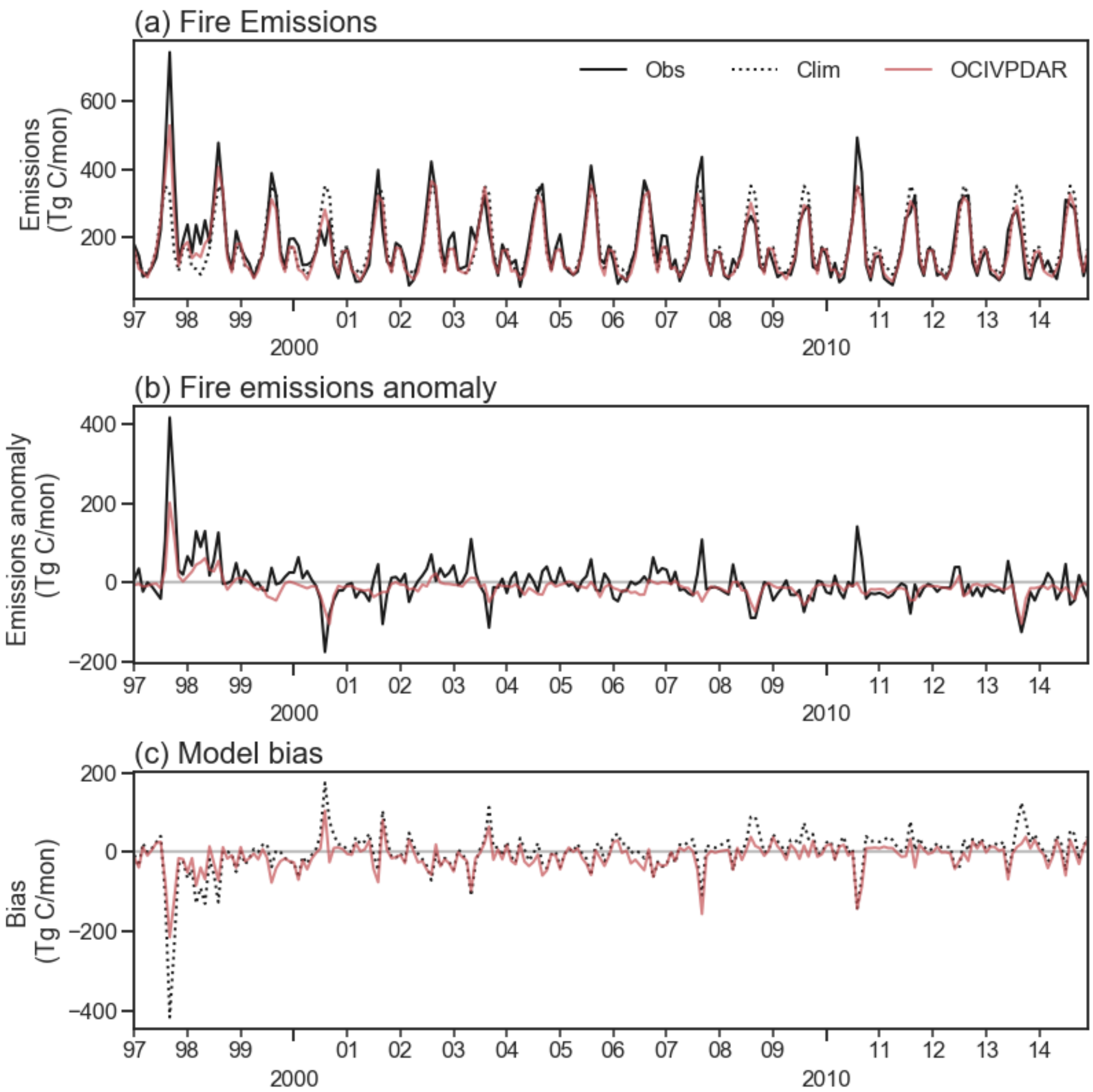

Figure S10. In-sample comparison of predicted values (global sum) with observations for the model development period (1997-2014). Model parameters were derived using all data in the training period. The 1-step results from the *OCIVPDAR* model with a 1-month lead time are shown.

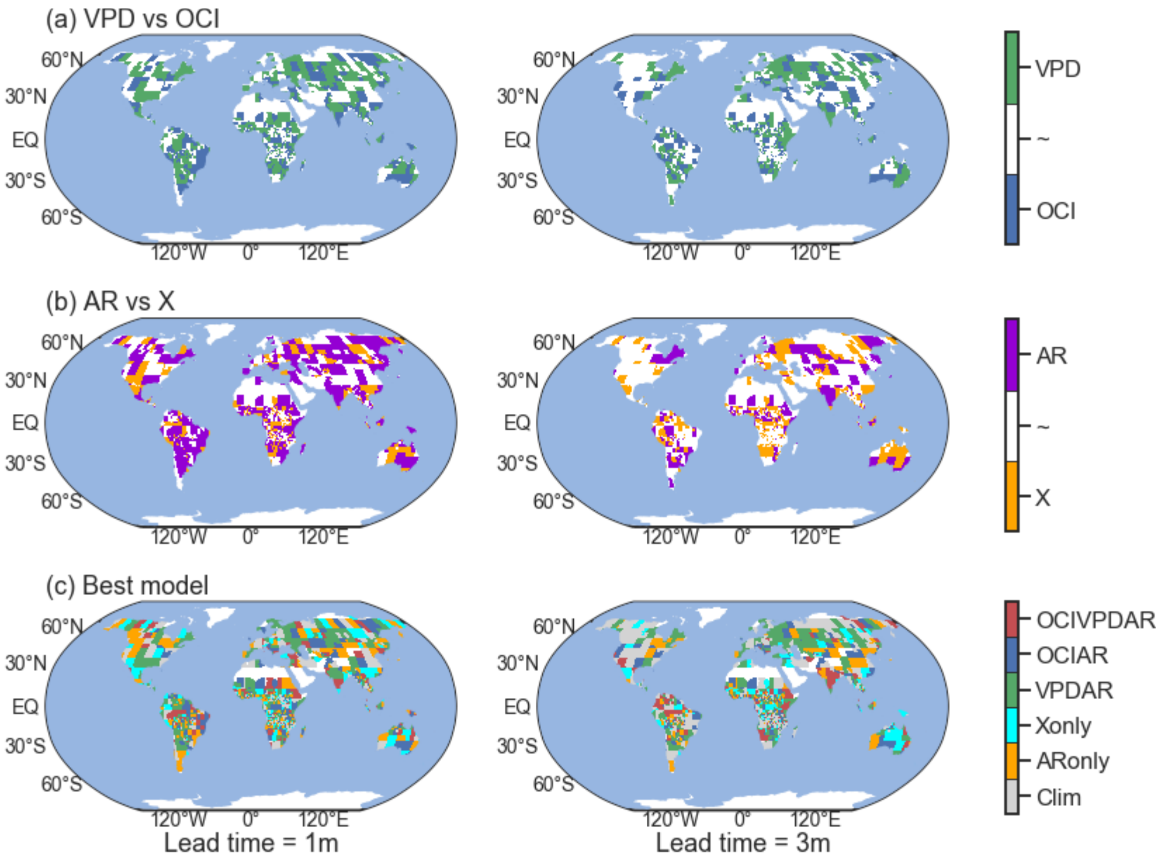

Figure S11. Global maps of (a) relative importance of VPD and OCI, (b) relative importance of autoregression (AR) and climate impacts (X), (c) the model with lowest RMSE in each FCR. In (a) and (b), the relative importance is determined by the RMSE comparison between the *VPDAR* and *OCIAR* models, and between *ARonly* and *Xonly* models, respectively. The FCRs in which model results are not significantly better than the *Clim* model in performance (<2% decrease in RMSE) are shown in white.
